# A generative adversarial network for synthetization of regions of interest based on digital mammograms

**DOI:** 10.1038/s41598-022-09929-9

**Published:** 2022-04-13

**Authors:** Olaide N. Oyelade, Absalom E. Ezugwu, Mubarak S. Almutairi, Apu Kumar Saha, Laith Abualigah, Haruna Chiroma

**Affiliations:** 1 School of Mathematics, Statistics, and Computer Science, University of KwaZulu-Natal, King Edward Avenue, Pietermaritzburg Campus, Pietermaritzburg, 3201 KwaZulu-Natal South Africa; 2 University of Hafr Al Batin, College of Computer Science and Engineering, Hafar Al Batin, Saudi Arabia; 3 Department of Mathematics, National Institute of Technology Agartala, Agartala, India; 4 Faculty of Computer Sciences and Informatics, Amman Arab University, Amman, 11953 Jordan; 5 School of Computer Sciences, Universiti Sains Malaysia, 11800 Gelugor, Pulau Pinang Malaysia

**Keywords:** Biotechnology, Cancer, Computational biology and bioinformatics, Mathematics and computing

## Abstract

Deep learning (DL) models are becoming pervasive and applicable to computer vision, image processing, and synthesis problems. The performance of these models is often improved through architectural configuration, tweaks, the use of enormous training data, and skillful selection of hyperparameters. The application of deep learning models to medical image processing has yielded interesting performance, capable of correctly detecting abnormalities in medical digital images, making them surpass human physicians. However, advancing research in this domain largely relies on the availability of training datasets. These datasets are sometimes not publicly accessible, insufficient for training, and may also be characterized by a class imbalance among samples. As a result, inadequate training samples and difficulty in accessing new datasets for training deep learning models limit performance and research into new domains. Hence, generative adversarial networks (GANs) have been proposed to mediate this gap by synthesizing data similar to real sample images. However, we observed that benchmark datasets with regions of interest (ROIs) for characterizing abnormalities in breast cancer using digital mammography do not contain sufficient data with a fair distribution of all cases of abnormalities. For instance, the architectural distortion and breast asymmetry in digital mammograms are sparsely distributed across most publicly available datasets. This paper proposes a GAN model, named ROImammoGAN, which synthesizes ROI-based digital mammograms. Our approach involves the design of a GAN model consisting of both a generator and a discriminator to learn a hierarchy of representations for abnormalities in digital mammograms. Attention is given to architectural distortion, asymmetry, mass, and microcalcification abnormalities so that training distinctively learns the features of each abnormality and generates sufficient images for each category. The proposed GAN model was applied to MIAS datasets, and the performance evaluation yielded a competitive accuracy for the synthesized samples. In addition, the quality of the images generated was also evaluated using PSNR, SSIM, FSIM, BRISQUE, PQUE, NIQUE, FID, and geometry scores. The results showed that ROImammoGAN performed competitively with state-of-the-art GANs. The outcome of this study is a model for augmenting CNN models with ROI-centric image samples for the characterization of abnormalities in breast images.

## Introduction

Deep learning (DL) models represent a more complex architecture of multilayer perceptron or artificial neural networks and are able to learn hidden patterns in data. It also represents a subfield of machine learning and draws its concept of architecture layout from the human brain. DL models consist of deep discriminatory models that map a high-dimensional, rich input to a class label, such as a digital image. These models have attained huge success in areas requiring complex input in a representation such as an image, pixels, document of text data, and files of audio and video data. Two popular variants of the DL model are the convolution neural network (CNN) and recurrent neural network (RNN). CNN is a type of DL that uses convolutional operation, pooling, fully connected, and activation functions to extract features from input data across different hierarchies for feature detection. CNNs have recorded noteworthy success in numerous fields, such as image processing, computer vision, and natural language processing and speech recognition^1–4^. While training a CNN model, it is often desired that loss values drop while the accuracy improves. When the fully trained model is exposed to the test dataset, an appreciable drop in loss and an increase in accuracy are also expected. However, insufficient training data may impair this performance, leading to overfitting or poor generalization^5^. Despite the use of dropout regularization, batch normalization, transfer learning, and other regularization techniques, the other limitations encountered provide intriguing research opportunities. To address these issues, data augmentation, a regularization technique, has proven useful and effective in mitigating the problem^6–8^. The data augmentation technique allows for artificially generating additional data from the available data by applying two approaches, namely, the standard or data transformation approach, neural style transfer, meta-learning, adversarial training, and the approach of generative adversarial networks (GANs). Transformation operations consist of image rotation, whitening, flipping, color space, cropping, translation, and noise injection.

**Figure 1:**
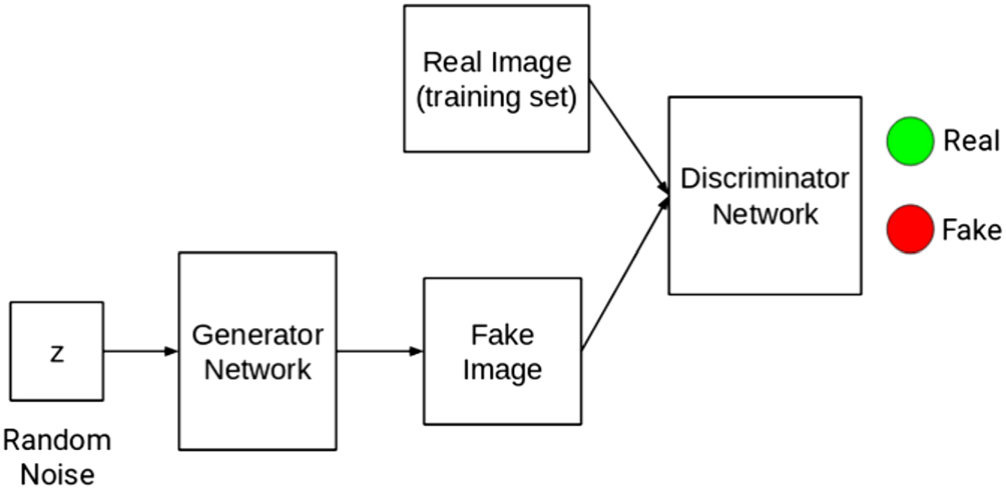
Illustration of the generator and discriminator^11^.

GANs are a type of DL with significant recent progress compared to DL itself. GANs operate by the adversarial positioning of two CNN models against each other. The two CNN models are the discriminator (D) and generators (G). D is tasked with detecting the probability of knowing if the output of G is from the model distribution or the data distribution, while G is required to synthesize images from noise. The implication of this adversarial arrangement is to enable D to estimate the probability that a sample image came from the training data rather than images generated by G. The combination of these models illustrates a minimax two-player game such that the training of G maximizes the probability of D making a mistake^9^. Measuring the loss of D will allow for quantification of how its performance is progressing, while the loss of G is aimed at quantifying its ability to trick D. Therefore, it is usually desired that G should perform well in a manner that its outputs are easily classified by D as real and not fake, as illustrated in Fig. 1. The resulting adversarial network, GAN, has demonstrated huge capability for data generation and has gained much attention in image classification and proven relevant in domains such as adaptation, data augmentation, and image-to-image translation^10^.

Data augmentation using GANs has been recognized to boost the sensitivity of diagnosis with a clinically acceptable number of additional false positives^12^. The popularity and adaptability of GANs is spurred on by challenges associated with accessibility to large amounts of medical images^13^ and a high level of restriction to some specific categories of medical images^14^. Another challenge of the publicly available dataset, which has now paved the way for wide adoption of GANs, is due to little or no availability of annotated data that medical experts have annotated^12,15,16^. Successive application of GANs as measures for mitigating the problem of overfitting in learning models has gained huge popularity among the community of deep learning researchers, especially those interested in image classification. It has also been observed that GAN models are relevant in domains other than image classification. Some examples are image-to-image translation, which is often applied to translating satellite photographs to Google Maps or converting day photos into night photos^17,18^; face aging and deaging^19,20^; attention prediction^21^; text-to-image translation^22^; generating realistic photographs^23,24^; generating cartoon characters^25,26^; face frontal view generation^27,28^; generating new human poses^29,30^; photos to emojis^31,32^; photograph editing^33,34^; photoblending^35^; superresolution^36^; photopainting^37–39^; clothing translation^40–42^; video prediction^43,44^; and 3D object generation^45–47^. These wide ranges of adaptability of GANs show that they have demonstrated excellent and broadly accepted performance in image generation.

However, GANs suffer from a few challenges, which still impede their constructability, and their adaptation to some real-world problems, such as medical sciences, remains a challenge^48^. In general, considering that the cross-domain applicability of GANs is problems such as instability of GAN models^49^, low quality or a substandard image generated by GAN models usually leads to low-resolution medical images and may affect the image performance of the classifier. Additionally, difficulty generating wide categories of images across domains is a notable setback. GANs also present challenging training due to their difficulty adapting their parameters for image generation across fields. Other related problems include insufficient high standard metrics for performance measurement^49,50^; mode collapse and GANs failing to converge^51^; overfitting resulting from imbalance from the D and G in GAN^52^; and trustworthiness of generated medical data, which may be subjected to ethical considerations, thereby limiting acceptability^50^. Most of these problems are researched through an architectural perspective or loss of the GAN model. In this study, we aim to remedy some of these well-known GAN problems and, importantly, generate ROI-based image samples to overcome problems of whole images in CNNs^53^.

The current study aims to increase the availability of ROI-based artificial synthesized digital mammograms for the characterization of abnormalities in digital breast images through the use of GANs. To achieve these goals, we propose ROImammoGAN, which is discussed in the subsequent sections. Therefore, we seek to propose procedures for improving the training of GANs to generate digital images of the breast. As a result of the proposed enhancement on our trained GAN, this study will further attempt to generate high-quality digital mammograms that deep learning models targeted at characterizing abnormalities in breast images and can employ for augmenting insufficient datasets. Our contributions as listed below:

- Designed G and D architectures resulting in a GAN model aimed at generating ROI-based digital mammograms
- We adapted the GAN model to generate sample images for different abnormalities, namely, architectural distortion, microcalcification, asymmetry, and mass.
- We evaluated the performance of the proposed GAN model in comparison with other similar state-of-the-art models.

The remainder of this paper is organized as follows: “Related works” section describes advances made in the design and implementation of GANs and the application of GANs; in “Overview of generative adversarial networks (GANs)” section, we provide an overview of basic concepts and terms used in GANs; in “Methodology for ROImammoGAN” section, the proposed GAN model is described in detail; "Experimentation" section details various experiments carried out; “Results and discussion” section presents the performance comparison of the proposed ROImammoGAN model with other state-of-the-art GANs. Finally, the paper is concluded in “Conclusion” section.

## Related works

In this section, we present the studies on generative adversarial networks and their applications in medical imaging. The review of studies on the application of GANs is limited to medical images, with particular interest in breast imaging—digital mammograms. Our approach to the review presented in this section assumes a chronological pattern.

### Generative adversarial networks (GAN)

Chen et al*.* described an information-theoretic extension to the generative adversarial network, namely, InfoGAN, which could maximize the mutual information between a small subset of the latent variables and the observation. The proposed InfoGAN model learned disentangled representations completely unsupervised and outperformed supervised learning approaches in similar tasks. The authors claimed that InfoGAN was successfully applied to disentangle writing styles from digit shapes on the MNIST dataset, pose from the lighting of 3D rendered images, hair style recognition, appearance of eyeglasses, and emotions. The study first compared InfoGAN with prior approaches on relatively clean datasets and then showed that InfoGAN could learn interpretable representations on complex datasets, where no previous unsupervised approach was known to learn representations of comparable quality^54^. This impressive performance of InfoGAN has yet to record significant success in medical imaging synthesis and feature extraction.

Another interesting variant of GAN applied to generating images from text is the StackGAN achieved through the stacking of two GANs on each other, as seen in the work of Zhang et al*.*^55^*.* Photorrealistic images were synthesized from text descriptions through their proposed stacked generative adversarial networks (Stack-GAN) to generate photorealistic images conditioned on text descriptions. The generative model, which has two stages, works thus: The Stage-I GAN sketches the primitive shape and basic colors, while the Stage-II GAN takes Stage-I results and text descriptions as inputs and generates high-resolution images. The two stages largely rely on the text description from which the images are being generated. We are particularly interested in the ability of StackGAN to generate high-resolution images using this two-stage approach, which may provide rich concepts to GAN models aimed at medial images. To make this generative model impressive, the authors reported that the model generates realistic 128 × 128 and 256 × 256 images (using the CUB and Oxford-102 datasets) with the Stage-II GAN able to rectify defects and add compelling details with the refinement process^55^. Wang and Gupta approached the design of their GAN by considering the basic formation of images consisting of a structure, which is the underlying 3D model, and style representing the texture mapped onto the structure. The resulting model named Style and Structure Generative Adversarial Network (S2-GAN) has the Structure-GAN that generates a normal surface map, and the Style-GAN takes the normal surface map as input and generates the 2D image. The authors revealed that they trained the two GANs independently and then merged them via joint learning, which they claimed can be used to learn unsupervised RGBD representations^56^. This approach is partly similar to the two-stage model demonstrated by StackGAN and will also present some traits that the digital mammogram generating model may likewise exploit.

The work of Nguyen et al.^57^ improved generative models for synthesizing realistic and high-resolution images through the performance of gradient ascent in the latent space of a generator network and introducing an additional prior on the latent code. These two additions to the generator network improve the sample quality and sample diversity and maximize the activations of one or multiple neurons in a separate classifier network. In addition, they provided a unified probabilistic interpretation of related activation maximization methods named plug and play generative networks (PPGNs), consisting of a generator network G that draws a wide range of image types and a conditioning network that tells the generator what to draw. The resulting GAN model successfully generated high-quality images at higher resolutions of 227 × 227 using the ImageNet dataset. They further applied their generative model to image painting and claimed that the proposed model could be applied to many types of data, including digital mammograms^57^.

The proliferation of research in GANs has led researchers to reconsider new loss functions for optimizing the performance of GANs. The Wasserstein GAN (WGAN) was an offshoot from this consideration and aimed to analyze the different ways to measure distances between the model distribution and the real distribution^58^. As a result, studies such as that of Arjovsky et al*.*^59^ targeted improving the learning stability of GANs and overcoming mode collapse introduced WGAN, an alternative to traditional GAN training. This concept proved relevant because it provided a meaningful learning curve useful for debugging and hyperparameter searches^59^. In another related work, Arjovsky et al*.* observed that although WGANs proved to attain stability during the process, they still generated only poor samples or failed to converge due to weight clipping in WGANs to enforce a Lipschitz constraint on the critic. Gulrajani et al*.*^60^ proposed penalizing the norm of the gradient of the critic concerning its input to circumvent the challenge of clipping weights. The result of this improvement is the production of high-quality images, and it enables stable training of a wide variety of GAN architectures with almost no hyperparameter tuning, including 101-layer ResNets using CIFAR-10 and LSUN bedrooms^60^.

Another study aimed to curtail the vanishing gradients problem during the learning process by reconsidering the use of a sigmoid cross-entropy loss function in the discriminator of regular GANs. The authors achieved this by proposing least squares generative adversarial networks (LSGANs), which adopt the least squares loss function for the discriminator, which translates into a minimization of the Pearson (math processing error) divergence. The benefit of this approach is the production of high-quality images compared to regular GANs and a more stable model during the learning process. The model is attractive due to its stability and generation of high-resolution images as required for medical images since their evaluation of LSGANs^61^.

In an attempt to manage the difficulty of training GANs and minimize the problem of mode collapse, Tolstikhin et al. A study proposed an iterative procedure where a new component is added into a mixture model at every step running a GAN algorithm on a reweighted sample. The resulting model was named AdaGAN and works by identifying potentially weak individual predictors and greedily aggregating them to form a robust composite predictor. The authors claimed that by this iterative and incremental procedure approach, their model attained convergence to the true distribution in a finite number of steps when each step was optimal and, as a result, addressed the problem of missing modes^62^. In addition to tackling training problems associated with GANs, Odena et al*.*^63^ constructed a variant of GANs employing label conditioning that results in generating 128 × 128 images of good resolution and global coherence. In addition, their conditional GAN was able to address the challenge of mode collapse, as they claimed that 84.7% of the classes have samples exhibiting diversity comparable to real ImageNet data^63^.

Pana et al*.*^64^ addressed the problem of ineffective saliency prediction by proposing a saliency GAN (SalGAN). SalGAN was designed as a data-driven metric-based saliency prediction method and trained with an adversarial loss function, having a two-stage approach to saliency prediction: the first one predicts saliency maps from the raw pixels of an input image; the second one takes the output of the first one to discriminate whether a saliency map is a predicted one or ground truth. The resulting SalGAN generated saliency maps that resemble the ground truth^64^.

Another study focused on improving the resolution of generated images from GANs and called the model superresolution generative adversarial network (SRGAN). The SRGAN consists of three components: network architecture, adversarial loss, and perceptual loss, which were further improved by Wang et al*.*^65^ to obtain an enhanced SRGAN (ESRGAN). The authors introduced the residual-in-residual dense block (RRDB) without batch normalization as the primary network building unit and allowed the discriminator to predict relative realness instead of the absolute value. The result of the ESRGAN showed that the model consistently outputs better visual quality and realistic images with more realistic and natural textures compared to SRGAN^65^. Furthermore, Wu et al*.* proposed a class-conditional GAN that performed contextual in-filling for synthesizing lesions on to healthy screening mammograms. Their GAN model, named ciGAN, was acclaimed to be able to generate high-resolution synthetic mammogram patches, with some diverse set of synthetic image patches at a high resolution of 256 × 256 pixels. The resulting ciGAN model was then applied to GAN-based augmentation, which improved mammogram patch-based classification by 0.014 AUC over the baseline model and 0.009 AUC over traditional augmentation techniques^66^. This study demonstrated a very close concept proposed in this paper, which seeks to synthesize patches of images for use in GAN-based augmentation in characterizing abnormalities in digital breast images.

Furthermore, Guibas et al*.*^67^ proposed a novel, two-stage pipeline for generating synthetic medical images from a pair of generative adversarial networks, tested in practice on retinal fundi images. The authors developed a hierarchical generation process to divide the complex image generation task into geometry and photorealism^67^. Similarly, Kazuhiro et al*.*^14^ also applied a deep convolutional GAN (DCGAN) model to generate human brain magnetic resonance (MR) images. They reported that the likelihood that images were DCGAN-created versus acquired was evaluated by 5 radiologists (2 neuroradiologists [NRs], vs 3 nonneuroradiologists [NNRs]) in a binary fashion to identify real vs created images. Images were selected randomly from the data set (variation of created images, 40–60%). None of the investigated images was rated as unknown. The NRs rated 45% and 71% as real magnetic resonance imaging images of the created images (NNRs, 24%, 40%, and 44%). In contrast, 44% and 70% of the real images were rated as generated images by NRs (NNRs, 10%, 17%, and 27%). The accuracy for the NRs was 0.55 and 0.30 (NNRs, 0.83, 0.72, and 0.64)^14^.

More attention was given to high-resolution images in GAN-based studies in 2018–2019. One such study produced BigGAN, which was proposed by Brock et al*.*^69^ to generate images of high-quality resolution. Their proposed GAN model was a class-conditional image generating model that works by applying orthogonal regularization to the generator, which renders it amenable to a simple “truncation trick”. This, therefore, allows fine control over the trade-off between sample fidelity and variety by reducing the variance of the generator’s input. The resulting BigGAN was successfully applied to generate high-resolution images of sizes 128 × 128, 256 × 256 and 512 × 512 using the ImageNet dataset. The authors claimed that the model outperformed similar models by obtaining an inception score (IS) of 166.5 and Fr ´echet inception distance (FID) of 7.4, improving over the previous best IS of 52.52 and FID of 18.65, and improved the state-of-the-art inception score (IS) and Fr ´echet inception distance (FID) from 52.52 and 18.65 to 166.5 and 7.4, respectively. In addition, at 256 × 256 and 512 × 512 resolutions, BigGAN achieved IS and FIDs of 232.5 and 8.1 at 256 × 256 and IS and FIDs of 241.5 and 11.5 at 512 × 512^68^.

Another study revealed that conditional GANs are now at the forefront of natural image synthesis and therefore attempt to concentrate on addressing these problems. The authors leverage two unsupervised learning techniques, adversarial training, and self-supervision, and take steps toward bridging the gap between conditional and unconditional GANs. They achieved this by allowing the three categories of networks to collaborate on representation learning. While the adversarial network plays its popular role, self-supervision encourages the discriminator to learn meaningful feature representations that are not forgotten during training. The authors reported that the resulting GAN model yielded high-quality synthesized images^69^. While Chen et al. worked on conditional GANS, Shaham et al*.*^70^ focused on unconditional GAN, which they called SinGAN. They revealed that SinGAN can learn from a single natural image without an accompanying label. SinGAN was able to capture the internal distribution of patches within the image and generate high-quality images, overcoming the challenge of mode collapse. In addition, they reported that SinGAN contained a pyramid of fully convolutional GANs, each responsible for learning the patch distribution at a different scale of the image. Their model, therefore, allows generating new samples of arbitrary size and aspect ratio that have significant variability yet maintain both the global structure and the fine textures of the training image^70^.

Similarly, while addressing the issue of high-resolution images to explore the capability of GANs in high-resolution image blending tasks, Wu et al*.*^71^ also presented a GAN model called the Gaussian-Poisson generative adversarial network (GP-GAN) to leverage the strengths of the classical gradient-based approach and generative adversarial networks. The novelty of their approach lies in the use of the Gaussian-Poisson equation to formulate the high-resolution image blending problem—described as a joint optimization constrained by the gradient and color information. In addition, they applied a blending GAN to learn the mapping between the composite images and the well-blended images. The resulting GP-GAN model produced high-resolution, realistic images with fewer bleedings and unpleasant artifacts^71^. Although previously reported GANs were largely applied to domains outside medical image generation, Yi et al*.*^10^ described a GAN capable of generating medical images that can help explore and discover the underlying structure of training data and learning to generate new images^10^.

Recently, a study proposed a novel U-net-based GAN model for data augmentation that can realistically synthesize and remove lesions on mammograms. With self-attention and semisupervised learning components, the U-Net-based architecture can generate high-resolution images of size 256 × 256 pixels. The study reported a significant improvement in malignancy classification performance due to their proposed GAN model^72^. While this study demonstrates a high-level GAN-based solution to the problem of scanty digital breast images used for deep learning classification purposes, we argue that their approach lacks the means to demonstrate overcoming the mode collapse problem. In addition, we observed that the proposed model was focused on generating high-resolution images only of size 256 × 256 compared to those proposed by some models presented above. Meanwhile, we observed that most of the GAN models presented in the studies reviewed above are primarily applied to nonmedical-based images, except for the works of two authors, 2018 and 2020.

This paper, therefore, is focused on designing a GAN model capable of generating multiclass high-resolution images for digital mammograms covering all the categories of abnormalities known with breast cancer. Mammograms contain both contextual information indicative of the breast anatomy and a great level of fine detail indicative of the parenchymal pattern. Much high-frequency information makes it imperative for radiologists to view these images at high resolution. In Table 1, we present a summary of all the related works which have been reviewed in this section. Meanwhile, the approach and domain of application of each of the GAN models are highlighted. Each study is compared with what is proposed in our work.

**Table 1:** A summary of related studies, their approaches, and application as compared with what is obtained in this study.

Author and reference	Approach and domain of application	Comparison with this study
Chen et al.^54^	InfoGAN: based on unsupervised learning which maximizes mutual information in a small subset of latent variables. Applied to writing styles using MNIST	RoiMammoGAN: used semi-supervised learning. Applied to breast images from mammograms using MIAS
Zhang et al.^55^	StackGAN: stacked two GANs on each other. Applied to generating photorealistic images using CUB and Oxford-102 datasets	RoiMammoGAN: one GAN model sufficiently and accurately achieved our aim. Applied to breast images from mammograms using MIAS
Wang and Gupta^56^	S2-GAN: composes of a style and structure GANS. Applied for generating structure and style in 2D images	RoiMammoGAN: learns the pattern and structure of abnormalities in medical images. Applied to breast images from mammograms using MIAS
Nguyen et al.^57^	PPGNs: used probabilistic interpretation and performance gradient to generate realistic and high-resolution images	RoiMammoGAN: combined the Adam gradient algorithm and performance increment to generate images. Applied to breast images from mammograms using MIAS
Neff^58^	WGAN: improved loss function performance using Wasserstein	RoiMammoGAN: a combination of RELU and LeakyRELU were used for computing loss function
Arjovsky et al.^59^	WGAN: aimed to stabilize learning pattern and reducing mode collapse in GAN	RoiMammoGAN: architectural composition showed that this model overcome mode collapse
Gulrajani et al.^60^	Used a penalization mechanism for norm gradient to overcome clipping weights. Used CIFAR-10 and LSUN bedrooms	RoiMammoGAN: the challenge of clipping weights was eliminated in our model. Applied to breast images from mammograms using MIAS
Mao et al.^61^	LSGAN: least squares loss function was used to curtail the vanishing gradients problem	RoiMammoGAN: kernel sizes of $D$ and $G$ were intelligently selected through investigative experimentation to overcome vansing gradient problem
Ilya et al.^62^	AdaGAN: addition of component through iterative procedure to avoid training problem	RoiMammoGAN: to eliminate complexity of learning features during training, staged-class-based learning was applied
Odena et al.^63^	CGAN: uses label conditioning to generate high resolution images	RoiMammoGAN: adopted the label conditioning strategy in addition to label flipping
Pana et al*.*^64^	SalGAN: designed as a data-driven metric-based saliency prediction method and trained with an adversarial loss function	RoiMammoGAN: the concept of saliency map was not considered in the study
Wang et al*.*^65^	SRGAN: high resolution focused GAN model	RoiMammoGAN: also a high-resolution focused GAN model
Wu et al.^66^	ciGAN: used for contextual in-filling for synthesizing lesions. Applied to mammogram patches	RoiMammoGAN: uses the class label to condition the learning and training process. Applied to mammogram ROIs
Guibas et al*.*^67^	two-stage pipeline and pair-based GAN for medical image synthesis	RoiMammoGAN: one-stage and single-based GAN for breast cancer mammography image synthesis
Kazuhiro et al*.*^14^	DCGAN: based on deep convolutional. Applied to magnetic resonance (MR) images	RoiMammoGAN: based on deep convolutional-transpose network. Applied to breast images from mammograms using MIAS
Brock et al*.*^69^	BigGAN: class-conditioning and orthogonal regularization was used in the generator to achieve fidelity and variety	RoiMammoGAN: class-label guided approach was used
Chen et al.^69^	Combined conditional and unconditional GANs with adversarial training and self-supervision	RoiMammoGAN: based on conditional GAN with adversarial training and semi-supervision
Shaham et al*.*^70^	SinGAN: unconditional GAN capable of learning from a single natural image without an accompanying label	RoiMammoGAN: learns from batch of images using conditional GAN approach
Wu et al*.*^71^	GP-GAN: leverage the strengths of the classical gradient-based for GAN	RoiMammoGAN: Adam gradient-based approach was used
Yi et al*.*^10^	A GAN can help explore and discover the underlying structure of medical images	RoiMammoGAN: can detect the structure of abnormalities in a digital mammogram (medical images)
Wu et al*.*^72^	U-net-based GAN was designed to generate lesions on mammograms	RoiMammoGAN: was also designed to generate lesions on mammograms
Oyelade and Ezugwu^73^	ArchGAN: capable of synthesizing mammograms with only architectural distortion	RoiMammoGAN: an advanced model of the ArchGAN

In our recent study^73^, we demonstrated the use of a GAN model for synthesizing medical images with architectural distortion abnormality. The RoiMammoGAN proposed in this study is novel and has some has distinguishing features with shared similarities with mainstream GAN models. Although it shares the element of conditional GAN, it advances this approach to using class-label flipping mechanism as a form of image augmentation strategy. Also, rather than using the normal convolutional operation, the proposed GAN model is based on deep convolutional-transpose network. The architectural composition of the model helped overcome major limitations of GANs such as mode collapse. RoiMammoGAN seeks to eliminate complexity of learning features during training through the use of staged-class-based learning approach.

### Application of GANs to medical imaging classification

There are several applications of various GAN-based models to medical imaging classification problems. However, since the focus of this paper is not to apply the GAN-based model to the CNN-like model used for classification purposes, we have decided to present only a few works that have applied GAN-based models for such problems. Zhang et al*.*^13^ applied deep convolutional GANs (DCGANs), Wasserstein GANs (WGANs), and boundary equilibrium GANs (BEGANs) to develop a medical image synthesis model that generates images for convolutional neural networks (CNNs), which can capture feature representations that describe a high level of image semantic information. The authors revealed that the effectiveness of the generative network on the CNN model yielded an accuracy of 98.83%^13^. Though many studies have been done with recurrent neural networks (RNN)^74–76^, data-generating models based on such neural networks are yet to gain popularity.

In another study, the authors applied a GAN-based model that uses automatic bounding box annotation to CNN-based brain metastasis detection on 256 × 256 MR images^12^. Korkinof et al*.*^77^ also demonstrated the benefit of adopting GAN models in generating full field digital mammograms (FFDMs) at a high resolution of 1280 × 1024 pixels for medical images^77^. This study is very interesting to us because of their ability to adapt their GAN model to a very difficult domain by generating realistic, high-resolution medical images for full-field digital mammograms (FFDMs). This difficulty usually arises from the textural heterogeneity, fine structural details and specific tissue properties of FFDM.

In another study, the author proposed the application of a semisupervised generative adversarial network (GAN)-based method to predict binding affinity. While GAN-based networks were used for feature extraction and a regression network for prediction, this combination was used to learn protein drug features of both labeled and unlabeled data^78^. MedGAN builds upon recent advances in the field of generative adversarial networks (GANs) by merging the adversarial framework with a new combination of nonadversarial losses. The study utilized a discriminator network as a trainable feature extractor that penalizes the discrepancy between the translated medical images and the desired modalities. Moreover, style-transfer losses are utilized to match the textures and fine structures of the desired target images to the translated images^79^. Very recently, Singh and Raza^80^ observed that DCGAN, Laplacian GAN (LAPGAN), pix2pix, CycleGAN, and unsupervised image-to-image translation model (UNIT) GANs have gained popularity in the interpretation of medical images^80^. Rijken et al.^81^ proposed a similar GAN model for synthesizing digital mammograms and named it MammoGAN. The authors reported that images with high resolution were successfully produced using their model. Although the approach was similar to what was proposed in this study, we note that their work was mainly on whole images and not ROI-based images, as we propose in this study.

## Overview of generative adversarial networks (GANs)

In this section, preliminaries on GANs are presented. We emphasize the structure of vanilla GANs and some recent architectures with interesting performances. In addition, the challenges posed with training GANs and some useful techniques for circumventing these limitations are also discussed.

### Discriminator and generator

**Figure 2:**
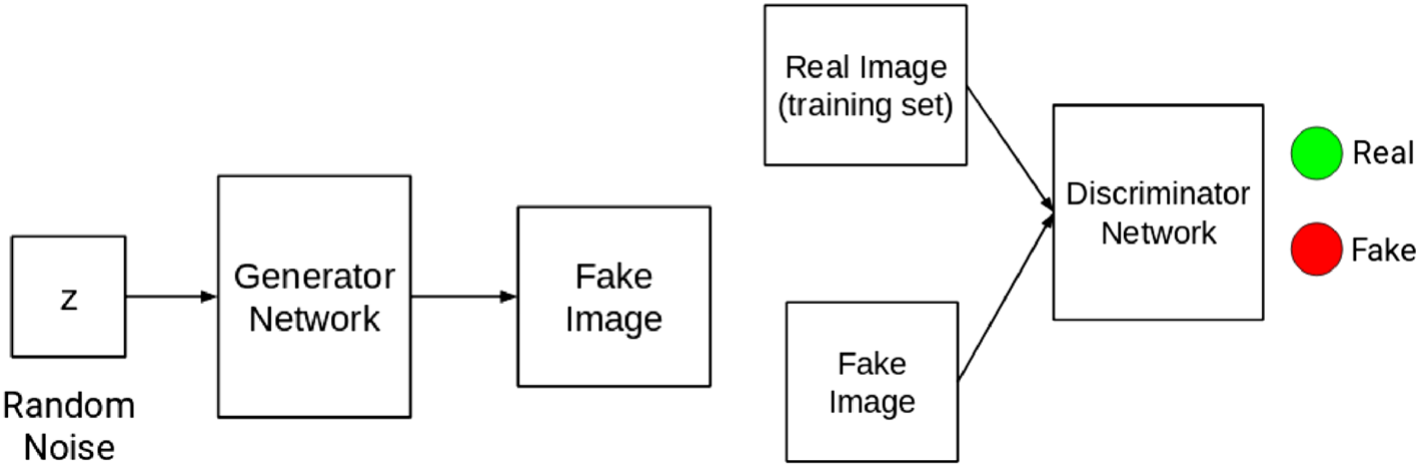
Illustration of generator and discriminator^11^.

The GAN model consists of the discriminator and generator. The generator, a deep network generator $\mathrm{G}$, is used to create an image $\mathrm{x }(\mathrm{x}=\mathrm{G}(\mathrm{z}))$ *from* a sample noise $\mathrm{z}$*,* drawn from using a normal or uniform distribution, is approximately equal to normal distribution say $N(\mathrm{0,1})$ or uniform distribution say $U(-1, 1)$. The generator $G$ is described as a differentiable function represented by a multilayer perceptron with parameters $\theta g$. The second component of the GAN model represents the discriminator $D$, which is also a multilayer perceptron $D(x;\theta d)$ that outputs a single scalar. $D$ performs a binary classification that classifies the input as real or fake by assigning 1 or 0. While G synthesizes images from random noise $z$, $D$ attempts to detect the originality or realistic level of the images fed into it. $G$ is tuned in such a manner to deceive $D$ to accept its generated images as real, while $D$ optimizes itself to detect fake images emanating from $G$ compared to real images drawn from a distribution of authentic images in a given domain. Figure 2 illustrates the composition of a generator $G$ and a discriminator $D$.

To learn the generator’s distribution $pg$ over data $x$, we define input noise variables $pz(z)$, then represent a mapping to data space as $G(z;\theta g)$, and train $D$ to maximize the probability of assigning the correct label to both training examples and samples from $G$. Therefore, both $D$ and $G$ are trained to outdo each for their different purposes: $G$ is aimed at minimizing $log(1-D(G(z)))$. Such training of $D$ and $G$ is described as a two-player minimax game with value function $V(G,D)$ so that the gradient information is back propagated from $D$ to $G$. By doing so, $G$ is able to correct its parameters in minimizing the possibility of $D$ recognizing its fake images. This results in a gameplay that eventually leads to either $D$ or $G$ winning and losing. The GAN model converges when the discriminator and the generator attain a situation when one player will not change its action regardless of the outcome of the opponent, something known as Nash equilibrium. We summarize this description in Eq. (1) from a study^9^. From Eq. (1) below, p(data) represents the distribution of data from the training set, $D(x)$ is the probability that x comes from the training data, and $p(z)$ represents the input noise variable.

1 $${\underset{G}{\mathrm{min}}}{\underset{D}{\mathrm{max}}}V(D, G)={E}_{x\sim pdata(x)}\left[\mathrm{log}D\left(x\right)\right]+{E}_{z\sim pz\left(z\right)}\left[\mathrm{log}\left(1- D\left(G\left(z\right)\right)\right)\right]$$

Equation (1) shows that the generator $G$ wants to minimize the function $D(G(z))$ to be 1, while the discriminator $D$ wants to maximize the function $D(x)$ to be close to 1 and $D(G(z))$ to be close to 0.

You will recall that one of the major problems addressed by some studies reviewed in “Generative adversarial networks (GAN)” section revealed that training GAN models can be very problematic. Here, we first illustrate how the training of GAN is carried out using Fig. 2.

**Figure 3:**
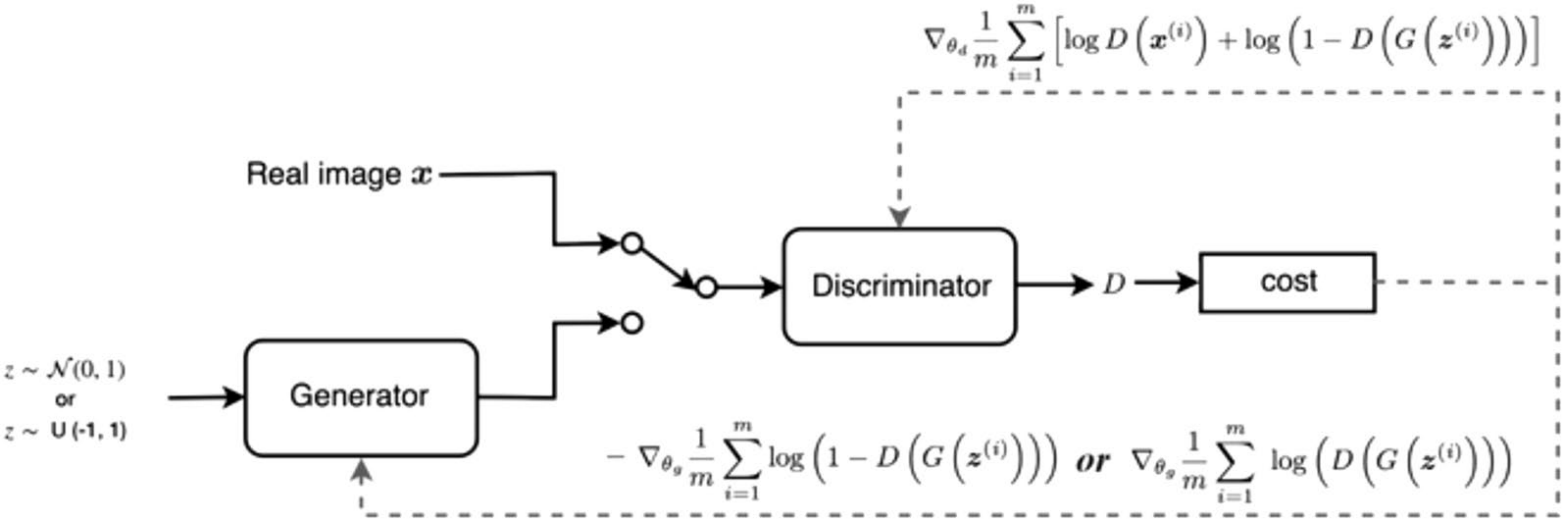
An illustration of how GAN is trained^52^.

In Fig. 3, input into G is drawn from either normal or uniform distribution, and the output of $G$ is passed to $D$ in addition to a real image drawn from a distribution of real data x. Additionally, the cost functions of both $G$ and $D$ are recursively updated for improved performance of the GAN model.

Image synthesis with GANs may assume the form of conditional or unconditional approaches. The conditional approach demands that additional information such as class labels be added to random noise generated from noise input distribution, while unconditional synthesis refers to image generation from random noise without any other conditional information. However, developers of GANs need to choose the appropriate technique for modeling their networks carefully. Techniques commonly adopted in the medical imaging community include DCGAN, WGAN, and PGGAN due to their good training stability.

### Selecting configuration parameters for GANs

We have pointed out in "Related works” section that one of the challenges of GANs is difficulty in training. Successful training will require choosing correct and appropriate parameters and hyperparameters to ensure that the desired GAN is not just stable but converges in reasonable time. Hence, it becomes necessary to know which parameters need to be fine-tuned during training. This also implies that every time the parameters of one of the models are updated, the nature of the optimization problem that is being solved is changed. Hence, it becomes necessary to select a model for minimizing errors (optimization) while guessing the calculation of parameters/weights of the GAN model. Different optimization flavors are used to minimize the loss errors, of which gradient descent (GD) is the most popular. Examples of GD optimization algorithms are stochastic gradient descent (SGD), Adadelta, RMSProp and Adam, although Adam appears to have gained the attention of GAN-based researchers^82^.

The hyperparameters that can be optimized in an optimizer such as Adam or SGD are learning rate, momentum, decay, beta1, beta2, and nesterov, activation functions at different layers (with sigmoid, tanh, ReLU, LeakyReLU, ELU, SeLU, and other activation functions widely used), batch size (experiment with values of 8, 16, 32, 54, or 128 for the batch size), loss functions (binary cross-entropy), the number of layers in G and D networks, the number of epochs (experiment with 100 epochs and gradually increase it to 1000–5000), and important hyperparameters named learning rate. The learning rate in GANs usually experiments with 0.1, 0.001, 0.0001, and other small learning rates.

When using the Adam optimization algorithm, the alpha, which is also referred to as the learning rate or step size, helps to control the weight at the end of each batch, while the momentum hyperparameter controls how much to let the previous update influence the current weight update. Additionally, when training GAN, it is widely observed that choosing a small learning rate, as small as 0.00001, slows the proportion that weights are updated, while larger values of 0.3 result in faster initial learning before the rate is updated. In addition, it is a normal practice to allow beta1 to be kept at 0.9, while beta2 is set to be at 0.999. The epsilon hyperparameter is usually used to avoid the vanishing gradient problem, resulting in division by zero, and it is sometimes set to 10E-8. Therefore, it is necessary for researchers to train GANs to carefully select parameters and hyperparameters to avoid some of the challenges associated with GAN stabilization.

### Challenges and techniques for stabilizing/optimizing GANs

There are three major challenges associated with GANs: model training instability, difficulty in performance evaluation, and mode collapse. GAN challenges present training problems because both the generator model and the discriminator model are trained simultaneously in a zero-sum game. Hence, this subsection attempts to summarize these problems and mitigation strategies in the literature.

A GAN is said to be stable when it converges in good time, and such a lack of convergence is the most common failure experienced in GAN training. Therefore, stabilizing and successfully optimizing GANs is a necessary technique to be acquired. GAN is said to converge when the discriminator cannot distinguish generated images from the generator and those from real images. Therefore, the problem of lack of convergence is indicative when the model loss does not settle down during the training process but exhibits a wavy pattern. Convergence failure is also described by a lack of attainment of equilibrium between the contesting networks $D$ and $G$. Another indicator of a lack of convergence is the zero-value attainment of discriminator loss close to zero and when the loss of the generator continues to rise beyond the bound. To overcome the lack of convergence, one could ensure the consideration of either very large kernel sizes or very small values in $D$ and $G$ and to ensure that the choice of optimization algorithm is appropriate and not aggressive.

Mode collapse is another common challenge encountered when training GANs. The mode collapse problem occurs when GANs generate images that lack some of the modes of the multimodal data it was trained on. For instance, training a GAN on a digital mammogram dataset yields only subcategory abnormalities of breast images if the model exhibits mode collapse. Another example of mode collapse is when a GAN trained on a dataset consisting of digits from 0 to 9 generates images of some of the digits^83^. The problem of mode collapse can therefore be summarized as when the distribution pg(x) learned by G focuses on a few limited modes of the data distribution pr(x) rather than generating diverse images from pr(x), thereby exhibiting low diversity of images with identical images pouring out of G. When training GAN, one can easily notice the presence of mode collapse if the line plot shows oscillations in the loss over time, especially in the generator model. Mode collapse is described as an architectural problem^84^ sometimes arising from their highly volatile nature and the use of the wrong hyperparameter and therefore may require some architectural adjustment to mitigate it. GAN training is highly volatile, and using other hyperparameters will lead to mode collapse or vanishing gradients.

There have been several attempts to address these problems of stabilizing GANs. The first solution is *AdaGAN, a network that uses* training a collection of generators instead of one generator^85^. Another network-based approach to tackle the nonstabilizing problem i*s MAD-GAN,* an approach using multiple generators and one discriminator that detects if the provided sample is real or fake and etects which generator was responsible for creating the fake sample^86^. Kashikar^84^ also provided a solution through a mechanism that allows the generator to recognize various possible data clusters/modes simultaneously without being limited to the task of fooling the discriminator^84^. Similarly, Mescheder et al*.*^87^ revealed that the main factors preventing convergence of GANS lie in the presence of eigenvalues of the Jacobian associated with gradient vector field with zero real-part and eigenvalues with a large imaginary part^87^. Hence, they adopted local convergence for simplified gradient penalties even if the generator and data distribution lie on lower-dimensional manifolds. In related work, they showed that such approaches are effective, as they use those to learn high-resolution generative image models for a variety of datasets with little hyperparameter tuning^88^.

In conclusion, a general observation states that a stable GAN will have a discriminator loss range between 0.5 and 0.8, while the generator loss may also range between 1.0 and 2.0 or even higher. In addition, the accuracy of the discriminator on both real and generated images may range between 50 and 90%. Once a GAN achieves this state of stability, it is expected that $G$ will be able to provide very high-quality images near the real images, thereby making it difficult for $D$ to spot the differences. However, when $D$ becomes too strong as opposed to $G$, the generated samples become too easy to separate from real samples, thus reaching a stage where gradients from $D$ approach zero and not guiding further training of $G$^10^. A word of caution: both $D$ and $G$ may likely start off erratic and move around a lot before the model converges to a stable equilibrium.

Other strategies for achieving stable GANs may include the use of batchnorms in $D$ and $G$; use of Tanh and ReLU activation functions in output and all layers of $G$ respectively while using LeakyReLU in all layers of $D$; using pooling layers with strided convolutions in $D$ and fractional-strided convolutions in $G$; normalization of images between − 1 and 1; the use of a modified loss function of max log $D$ instead of min $log (1-D$); application of dropouts in $G$ such as a dropout which can be applied on several layers of our generator at both training and test time^89^; flipping of labels when training $G$ such that real images is turned to fake while fake images is presented as real; deriving input to $G$ mostly from normal distribution; use of average pooling, Conv2d and stride for downsampling while ConvTranspose2d and stride may be used for upsampling; addition of noise to labels such that real image label is replaced with values between 0.7 and 1.1 while fake image label can be replaced with values between 0.0 and 0.4^90^; keep checkpoints from the past of $G$ and $D$ and occasionally swap them out for a few iterations; adopt Adam optimization algorithm for $G$ and sometimes SGD for $D$; observe when loss of $G$ is steadily decreasing while $D$ goes down to zero during training; and finally, consideration of adding an additional channels to images.

## Methodology for ROImammoGAN

In this section, we describe the approach used to design the proposed ROImammoGAN. First, the framework is discussed, and the generator and discriminator and the algorithm design are presented afterward. It is noteworthy stating that all methods were carried out in accordance with relevant guidelines and regulations as prescribed by the journal.

### ROImammoGAN framework

**Figure 4:**
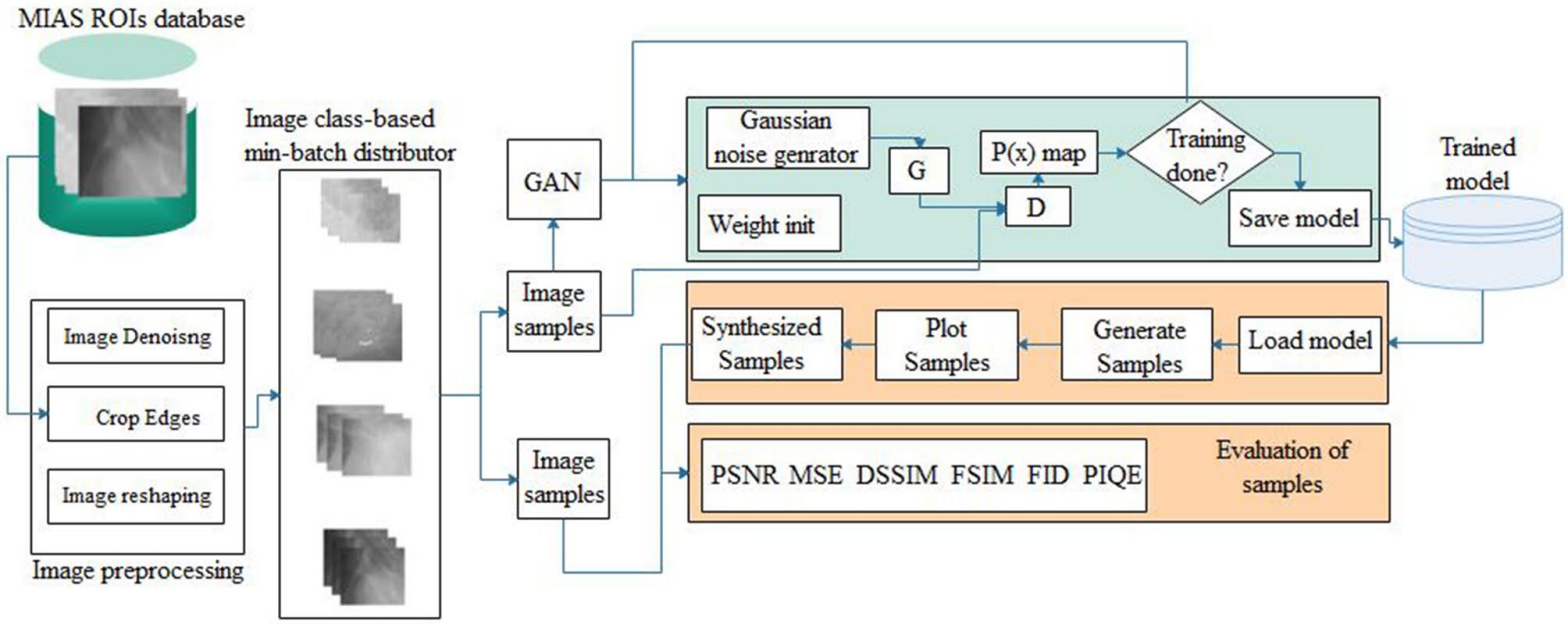
The proposed ROImammoGAN framework with the image preprocessing component and the GAN consisting of a generator and discriminator.

The framework that combines GAN architectures proposed in this study is presented in Fig. 4. The framework consists of a preprocessing image module, the generator $G,$ and discriminator $D$. We applied the framework to generate images of different abnormalities of breast cancer in digital mammography. The proposed framework is supplied with input from a dataset **ⅆ** and then batched into **ß** batch size, resulting in **ℵ** groups of **ß**. Image samples are randomly drawn from four abnormality classes: architectural distortion, microclassification, asymmetry, and mass. To allow for training and fine-tuning the GAN model to the required peculiarity of each abnormality, the framework was designed to apply class-based samples iteratively. The implication of this is that only samples drawn from a particular class are applied to the GAN model during training. As a result, the proposed GAN model was fine-tuned for each class of abnormalities to promote good performance during image synthesis. Since the study aims to synthesize images with abnormalities, we omitted samples with normal (nonmalignant) labels, considering that such samples dominate in all datasets.

A batch of sample images in the group are first applied to image preprocessing techniques to allow for removing noise, cropped out unwanted edges, and resized input to support the fixed size allowed by the architecture. A batch consists of **ß** samples of ***WxH*** that are passed as input to $D$. Image preprocessing became necessary to reduce the high-resolution image sources from publicly accessible digital mammography databases. Hence, our original images from **ⅆ** are cropped into images of sizes **[128, 128, 1]** and resized to **[64, 64, 1]** without losing the quality of images from **ⅆ**. Furthermore, random numbers that serve as noise stored in $z$ and of size 100 are generated using a uniform distribution and then pass as input to $G$. The two adversarial architectures, D and $G$, are then trained for a given number of epochs ***E*** until an appropriate model is achieved, depending on the abnormality represented in the images. We save different representations of model $G$ that are able to synthesize classed-based images. Meanwhile, during training, the proposed framework outputs **ß** size of images generated by $G$ after a specified number of iterations. We observe the quality of images generated across the training phase, and as the generated images appear real with $D$ able to increase its classification accuracy, training is ended, and the state of the model is saved for the class of abnormality for which it has been trained.

### The generator (G) and discriminators (D)

**Figure 5:**
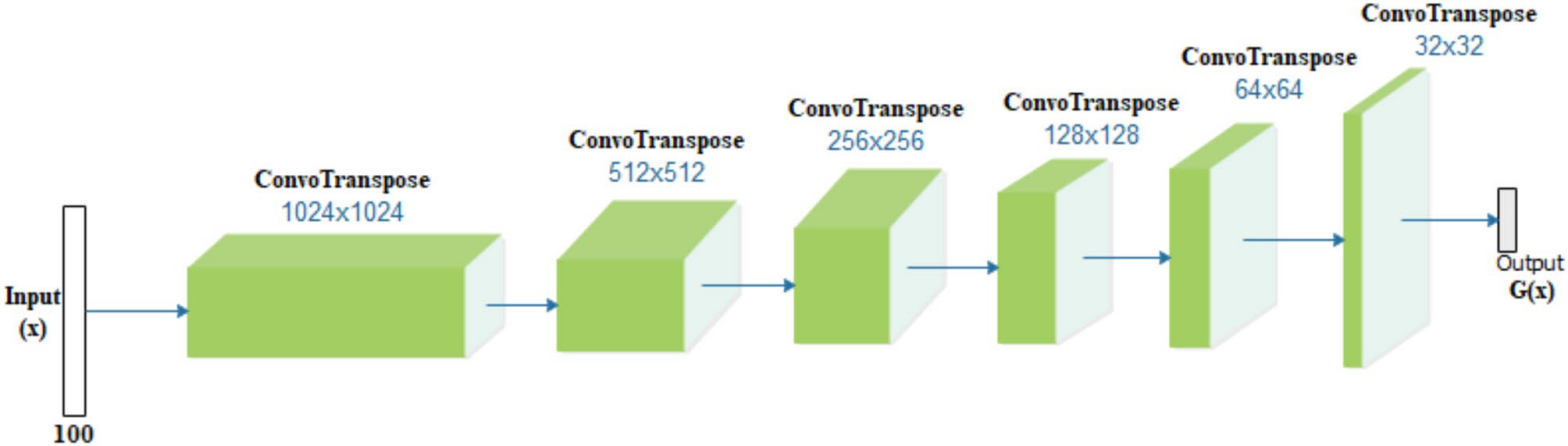
The generator (G) proposed in the ROImammoGAN and accepts input of size 100-dimension.

**Figure 6:**
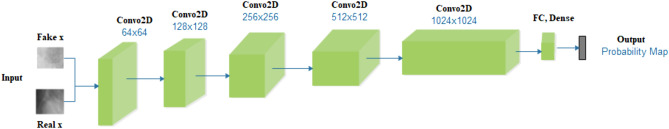
The discriminator ($D$) accepts inputs of either a real image from **ⅆ** or fake from G and outputs the probability [between 0.0 and 1.0], indicating when an input is either real or fake.

The GAN model combines generator $G$ and discriminator $D$ architectures represented in Figs. 5 and 6, respectively. Network G accepts input *z,* which is further reshaped to 4 × 4 × 512 and then forwards the resulting input through 6 blocks of transpose convolutional layers. Each layer applies a batch normalizer while using the ReLU activation function and a normal weight initializer with a standard deviation of 0.02. The generator G consists of a fully connected layer projecting input of a 100-dimensional uniform distribution to the network layers that use a 5 × 5 filter size. The discriminator network D consists of feature extractor *F* (*img*) and a layer for classification using sigmoid with weight vector ψ_*l*_. Input to D is scaled to the range of the tanh activation function [− 1, 1]. Additionally, D consists of five fractionally strided convolution layers and a sixth layer of flattening that is fully connected and applies a sigmoid activation function. Each layer of D is designed to use batch normalization except for the last layer. This has been applied to mitigate the problems that often arise from poor weight initialization and stabilize learning by normalizing the input to each unit to zero mean and unit variance. The layers of both G and D use a kernel size of 5 × 5 and filter count of 1024, 512, 256, 128, and 64 and those of 64, 128, 256, 512, and 1024 for G and D, respectively. Layer D uses leaky rectified linear unit functions with a slope of leak = 0.2; contrary to the use of RELU applied to G. for padding in both G and D, the same valu*e* was used in all layers of the two networks. A summary of the design of G and D is outlined in Tables 2 and 3, respectively.

**Table 2:** Generator architecture: we adopted the input noise vector of dimensionality 100 drawn from a zero-mean Gaussian distribution.

	Input projection	Layer1	Layer2	Layer3	Layer4	Layer5	Layer6
Type	Fully connected	Fractionally strided convolution	Fractionally strided convolution	Fractionally strided convolution	Fractionally strided convolution	Fractionally strided convolution	Fractionally strided convolution
Input	[1 × 100]	[4 × 4 × 1024]	[8 × 8 × 512]	[16 × 16 × 256]	[32 × 32 × 128]	[64 × 64 × 64]	[128 × 128 × 32]
Output	[4 × 4 × 1024]	[8 × 8 × 512]	[16 × 16 × 256]	[32 × 32 × 128]	[64 × 64 × 64]	[128 × 128 × 32]	[64 × 64 × 2]
Activation	ReLU	ReLU	ReLU	ReLU	ReLU	ReLU	TanH
Batch norm	Yes	Yes	Yes	Yes	Yes	Yes	Yes
Stride	–	2	2	2	2	1	–
Padding	–	same	Same	Same	Same	Same	Same
Kernel Size	–	5	5	5	5	5	5
Kernels	–	1024	512	256	128	64	32

Minibatch Size: 32, Optimizer: Adaptive Moment Estimation (Adam) (η = 0.00001, β1 = 0.5, β2 = 0.999). All weights were initialized using the normal distribution initializer.

**Table 3:** Discriminator architecture: Minibatch Size: 32; Optimizer: Adam (η = 0.0001, β1 = 0.5, β2 = 0.999).

	Layer1	Layer2	Layer3	Layer4	Layer5	Output
Type	Convolution	Convolution	Convolution	Convolution	Convolution	Full Con
Input	[32 × 32 × 2]	[64 × 64 × 64]	[32 × 32 × 128]	[16 × 16 × 256]	[8 × 8 × 512]	[4 × 4 × 1024]
Output	[64 × 64 × 64]	[32 × 32 × 128]	[16 × 16 × 256]	[8 × 8 × 512]	[4 × 4 × 1024]	[1]
Activation	LeakyReLU	LeakyReLU	LeakyReLU	LeakyReLU	LeakyReLU	Sigmoid
Batch norm	Yes	Yes	Yes	Yes	Yes	–
Stride	2	2	2	1	1	–
Padding	Same	Same	Same	Same	Same	–
Kernel Size	5	5	5	5	5	–
Kernels	64	128	256	512	1024	–

Considering that training GANs requires that a Nash equilibrium be found for a two-player noncooperative game, we experimented with a combination of SGD and Adam and purely used Adam optimization algorithms in $D$ and $G$. Finally, a gradient-based minimization technique was applied to minimize each player’s cost simultaneously. The models' weights in the proposed ROImammoGAN are initialized as we have experimented with the He initializer variance scaling initializer of type float32 and from a zero-centered normal distribution with a standard deviation of 0.02. A dropout of 0.5 was applied in D and G, and the performance was investigated using a batch size of 32.

### Algorithm of the GAN framework

In Algorithm 1, the procedure for the framework proposed in this study is outlined. Algorithm 2 first highlights how image processing techniques are applied to class-based inputs batched into the GAN model. Training of the proposed ROImammoGAN begins with $G$ receiving a random seed $(z)$ as input, which translates into an image $G(z)$. Afterward, $D$ is used to classify real images drawn from **ⅆ** and fake sample images generated from $G$. The trained model is then used to generate images during the testing phase for the class of images for which it is trained. In every iteration, the algorithm computes the loss of $D$ and $G$ to check whether the model is doing well or otherwise. In addition, the gradients of the loss values are used to update the generator and discriminator. Meanwhile, we evaluate the accuracy of the synthesized images generated by $G$ on every epoch.
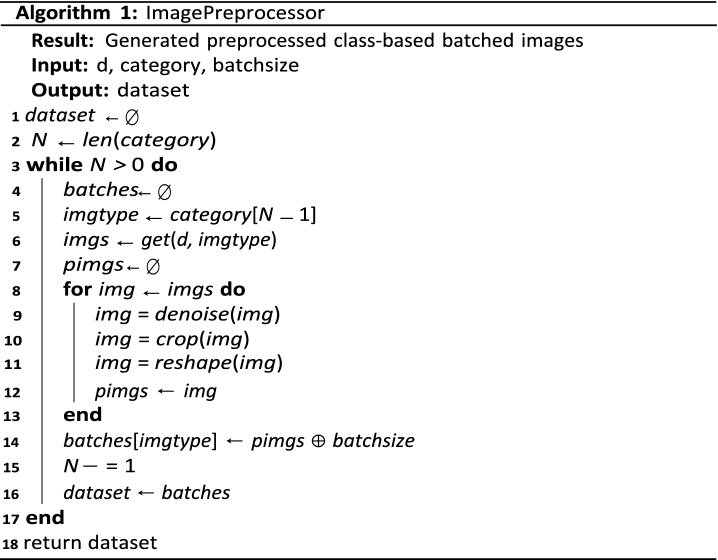

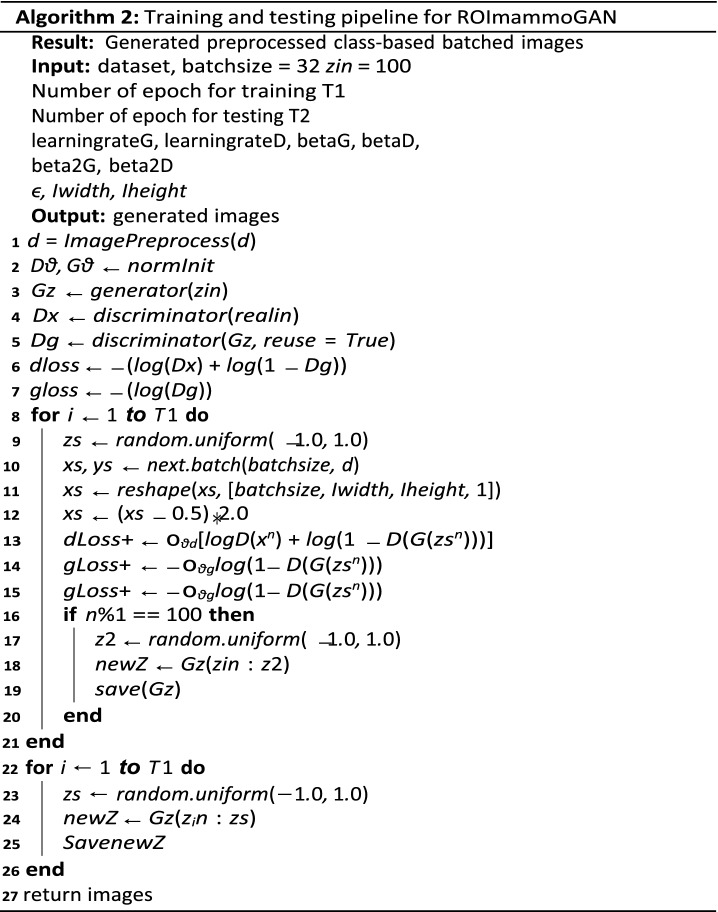


## Experimentation

This section presents different experiments carried out to achieve the state-of-the-art GAN model proposed and detailed in "Methodology for ROImammoGAN" section. First, we describe the training and testing parameter settings and the configuration of the computational resources utilized. Furthermore, we provide a detailed discussion of the datasets by presenting the metadata on the dataset and presenting a detailed illustration of the four (4) major abnormalities associated with breast cancer in digital mammography. Finally, to demonstrate the credibility of the performance of the proposed GAN model, we evaluated the outcome using gold standard metrics. These metrics are presented in detail, and the motivation for their selection has also been justified.

### Configuration experimentation environment and parameter setting

Training and testing were performed using the TensorFlow library and dependent libraries using Python 3.7.3. The computational environment consists of an Intel (R) Core i5-7500 CPU 3.40 GHz, 3.41 GHz; RAM of 16 GB; 64-bit Windows 10 OS. The experimentation carried out in this research demonstrates how the proposed ROImammoGAN is trained from scratch until good performance of image generation is achieved. We have investigated the performance of different parameters to understand how the model learns the problem well. For instance, for the learning rate hyperparameter, we experimented with 0.00001 and 0.0001 for G and 0.00004 and 0.0004 for D, respectively. The Adam optimizer was used for both G and D with parameter values set as beta = 0.5 and beta2 = 0.999 for $G$ and beta = 0.5 and beta2 = 0.999 for $D$. The value of 1e−08 was set for the Adam optimizer epsilon parameter with a batch size of 32. To improve the performance of our model, we applied a smoothening operation to the labels using a smooth factor of 0.1.

### Experimentation datasets

Most of the publicly available digital mammogram databases are the Mammographic Image Analysis Society (MIAS) database^91^, Digital Database for Screening Mammography (DDSM)^92^, INbreast database^93^, Breast Cancer Digital Repository (BCDR), and Image Retrieval in Medical Applications (IRMA). Table 4 summarizes the number of images taken from different databases for this study.

**Table 4:** Description of some benchmarked datasets used for experimentation.

Database	No. of patients	No. of images	Cases of abnormalities	Description
MIAS	161	322 (MLO view of images)	All forms of abnormalities (32 shows architectural distortion)	Digitised to 50 micron pixel edge, and reduced to is 200 micron pixel edge and padded/clipped so that all the images are 1024 × 1024
				Images include radiologist's truth-markings
DDSM	2620	10,480 (MLO and CC view of images)	All forms of abnormalities (approximately 137 shows architectural distortion)	The database has some associated patient information (like age at the time of study) and image information (like spatial resolution)
				Images are marked with ground truth information about the locations and types of suspicious regions

In this study, the MIAS datasets were used with a focus on the extraction of regions of interest (ROIs) for the whole image. We have focused on the details of the number of ROIs extracted both manually and using the automated method. In addition, statistics of the occurrence of the four different forms of abnormalities are presented. Samples derived as ROIs were resized to 299 × 299 pixels. The DDSM and CBIS-DDSM datasets have already been preprocessed and converted to 299 × 299 images by extracting the ROIs. Table 5 summarizes the description of samples from each of the databases. Our MIAS and DDSM + CBS ROI datasets are preprocessed into NumPy files, which are much more convenient to load and use in training.

**Table 5:** Description of datasets used for experimentation.

	Dataset	Total no. of samples/ROIs	No. of samples/ROIs with abnormalities
Benchmark dataset	DDSM + CBIS-DDSM	The dataset contains 55,890 of which 14% are positive and the remaining 86% negative	7824
	MIAS	5136 ROIs	536

**Figure 7:**
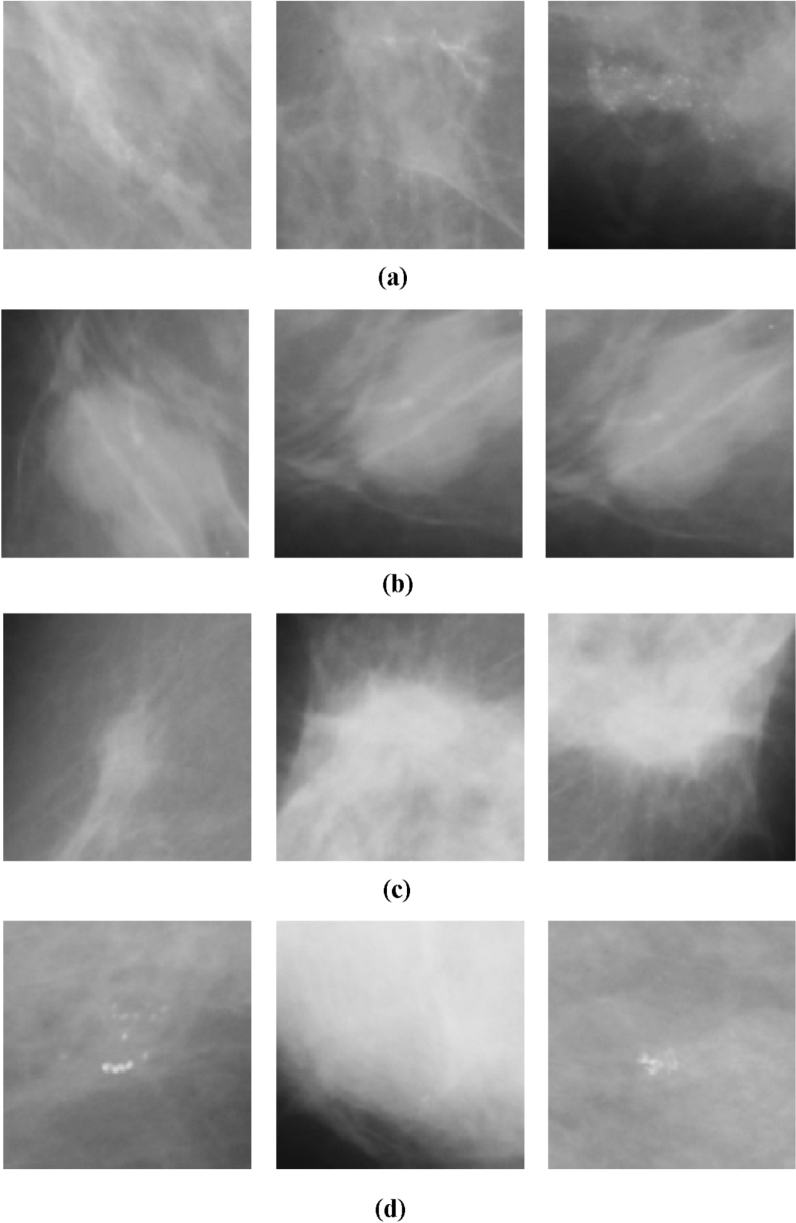
(**a**) Sample digital breast images with abnormalities characterized by architectural distortion from the MIAS dataset were drawn from a random batch of images during training. (**b**) Sample digital breast images with abnormalities characterized by asymmetry from the MIAS dataset were drawn from a random batch of images during training. (**c**) Sample digital breast images with abnormalities characterized by microcalcification from the MIAS dataset were drawn from a random batch of images during training. (**d**) Sample digital breast images with abnormalities characterized by mass from the MIAS dataset were drawn from a random batch of images during training.

We applied the selected image preprocessing techniques to the samples for uniformity in sizing and other features. Figure 7a–d outline the ROI format of samples extracted for the experimentation for the abnormalities, including architectural distortion, asymmetry, microcalcification, and mass. Samples are batched and resized and then serve as input to the discriminator.

### Performance evaluation metrics

The evaluation of the proposed GAN model was carried out using relevant image analysis metrics. These quantitative and qualitative metrics are grouped into feature-based, reference-based, and nonreference-based metrics. The metrics are applied to the images generated by the GAN generator after achieving optimal training. The evaluation aims to compute numerical scores to support presenting the summary of the quality of generated images. Meanwhile, we note that a wide range of metrics exists in the literature for evaluation which includes the following: average log-likelihood, coverage metric, inception score (IS), modified inception score (m-IS), mode score, AM score, maximum mean discrepancy (MMD), Wasserstein critic, birthday paradox test, classifier two-sample tests (C2ST), boundary distortion, number of statistically different bins (NDB), image retrieval performance, generative adversarial metric (GAM), tournament win rate and skill rating (TWRSR), adversarial accuracy and adversarial divergence, reconstruction error, sharpness difference, low-level image statistics (LLIS), Precision, Recall and F1 Score of generated images, universal image quality index (UIQI) and normalized relative discriminative score (NRDS).

In this study, the following quantitative techniques have been applied for evaluating the quality of images synthesized by the ROImammoGAN generator model: geometry score and Frechet inception distance (FID) as feature-based metrics; BRISQUE, NIQE, and PIQE as nonreference-based metrics and utilizes statistical features of a synthesized image to evaluate the image quality. Last, PSNR, FSIM, MES, DSSIM, and SSIM are the reference-based metrics used in this study to evaluate the quality of a synthesized image against the real image.

#### Feature-based metrics

##### Frechet inception distance (FID)

The FID metric is a candidate formula to compute the distance between the vector representation of the synthesized and real images. It is expected that a synthesized image with good quality should result in a lower FID score; otherwise, the image is assumed to be less similar to the real image. The highest similarity the two images may demonstrate is having an FID score of 0.0, maybe computed using Eq. (2).

2 $${d}^{2}\left(\left(m,C\right), \left({m}_{w}, {C}_{w}\right)\right)={\Vert m- {m}_{w}\Vert }_{2}^{2}+Tr(C + {C}_{w}-2{(C{C}_{w})}^{1/2})$$

##### Geometry score (GS)

The GS is another feature-based metric useful for comparing the real and synthesized images of GAN models. The metric computes its values using the variation in the geometrical properties of the real and synthesized images. The result may be used to measure the qualitative and quantitative values in evaluating the performance of the GAN model. The lower the value obtained in computing GS, shown in Eq. (3), the better the performance of the GAN model.

3 $$GS\left({X}_{1}, {X}_{2}\right)=\sum_{i=0}^{{i}_{max}-1}{(MRLT\left(i, 1, {X}_{1}\right) - MRLT(i, 1, {X}_{2}))}^{2}$$

#### Nonreference based metrics

##### Blind/referenceless image spatial quality evaluator (BRISQUE)

The BRISQUE is an image evaluation metric that is often referred to as *opinion-aware* and analyses images with similar distortions. The metric uses a subjective quality score.

##### Natural image quality evaluator (NIQE)

The NIQE metric, also referred to as *opinion-unaware*, computes the quality of images with arbitrary distortion. Contrary to BRISQUE, NIQE does not use subjective quality scores, and hence the assessment of the comparison of BRISQUE and NIQE may not be readily obvious from a mere look.

##### Perception-based image quality evaluator (PIQE)

Whereas both BRISQUE and NIQE require a trained model for their computation, PIQE does not. PIQE computes the quality of a given image based on an arbitrary distortion in a blockwise approach.

#### Reference metrics

##### Mean square error (MSE)

The MSE computes the average squared difference between the real and synthesized images and is computed using Eq. (4).

4 $$MSE(f,g)=\frac{1}{mn}\sum_{i=0}^{m-1}\sum_{j=0}^{n-1}{[f\left(i,j\right)-g(i, j)]}^{2}$$

##### Structured similarity indexing method (SSIM)

The SSIM, in Eq. (5), consists of three other metrics, namely, loss of correlation (LC) in Eq. (6), luminance distortion (LD) in Eq. (7) and contrast distortion (CD) in Eq. (8) to evaluate the quality of an image compared with another. The LC metric computes the correlation coefficient between the two images. CD evaluates the contrast of two given images using their standard deviation as it approaches 1 as the similarity of the images increases. LD computes the closeness of the mean luminance for two given images and may return a value close to 1 as the similarity of the images increases.

5 $$SSIM(f,g)=l\left(f,g\right)c\left(f,g\right)s(f,g)$$

6 $$l(x,y)=\frac{2{\mu }_{x}{\mu }_{y}+ {C}_{1}}{{\mu }_{x}^{2}+{\mu }_{y}^{2}+ {C}_{1}}$$

7 $$c(x,y)=\frac{2{\sigma }_{x}{\sigma }_{y}+ {C}_{2}}{{\sigma }_{x}^{2}+{\sigma }_{y}^{2}+ {C}_{2}}$$

8 $$s(x,y)=\frac{{\sigma }_{xy }+ {C}_{3}}{{\sigma }_{x}{\sigma }_{y}+ {C}_{3}}$$

##### Structural dissimilarity (DSSIM)

DSSIM is evaluated with SSIM and may be computed as shown in Eq. (9)

9 $$DSSIM(x,y)=\frac{1-SSIM(x,y)}{2}$$

##### Feature similarity indexing method (FSIM)

To obtain the normalized mean value of feature similarity between the real image and a corresponding synthesized image, we apply the FSIM, as shown in Eq. (10).

10 $$FSIM=\frac{{\sum }_{x \in \alpha }{S}_{L}x\left(x\right). {PC}_{m}(x)}{{\sum }_{x \in \alpha }{PC}_{m}(x)}$$

##### Peak signal to noise ratio (PSNR)

The PSNR measures the quality of an image with respect to a synthesized image by using MSE, as shown in Eq. (11). The higher the value of PSNR is, the more acceptable the quality of the image generated by the GAN model.

11 $$PSNR\left(f,g\right)=10{log}_{10} \left(\frac{{255}^{2}}{MSE(f,g)}\right)$$

Other interesting metrics have been applied in this study and presented in the next section, such as the loss and accuracy of generated images and the loss of both $D$ and $G$.

## Results and discussion

This section presents the result of the experimentation described in the last section and compares its performance with other similar models. We have further discussed the benefit of the proposed model and training techniques adopted to achieve a stable, converging, and nonfailing GAN model. We enabled the GAN model to train each category of abnormality for a long time until the generated images improved. As a result, the training epoch for each class of abnormalities is not the same. We graphed the loss and accuracy of the generator and discriminator during training and during image synthesis.

**Figure 8:**
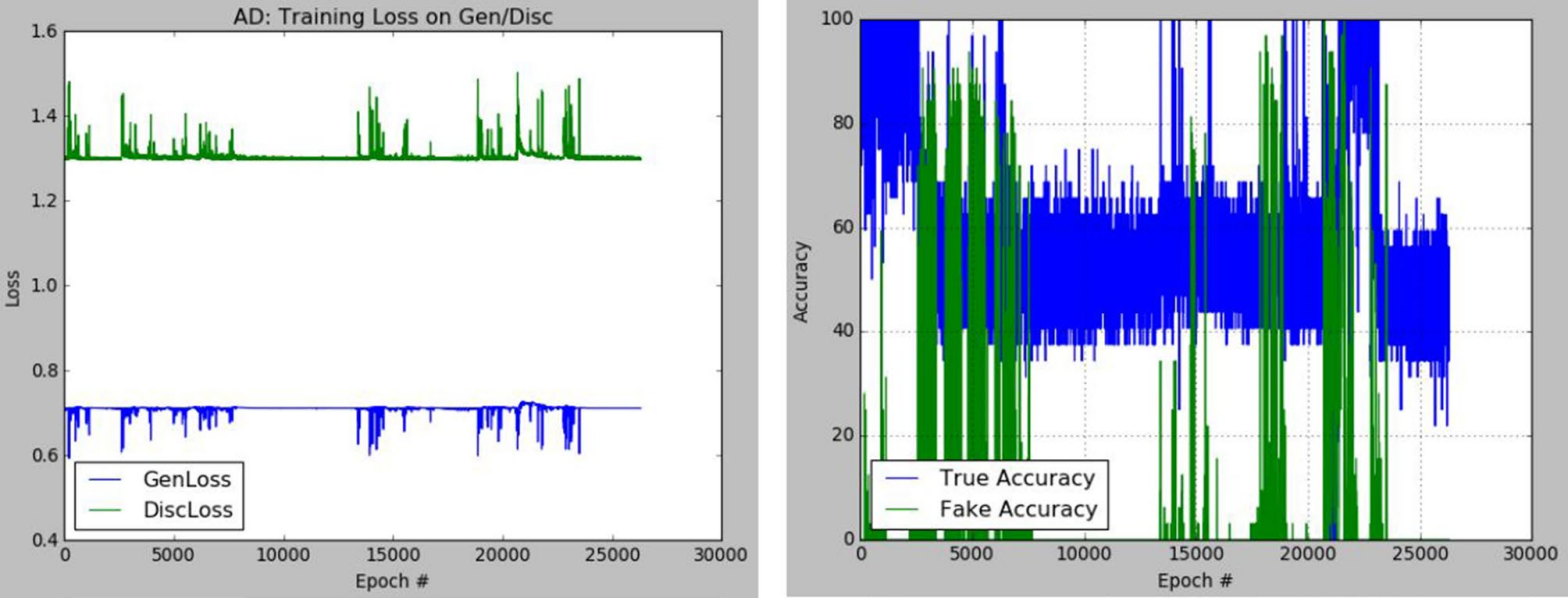
Training output on the proposed GAN model for architectural distortion showing how the generator learns in synthesizing images similar to real samples with architectural distortion.

**Figure 9:**
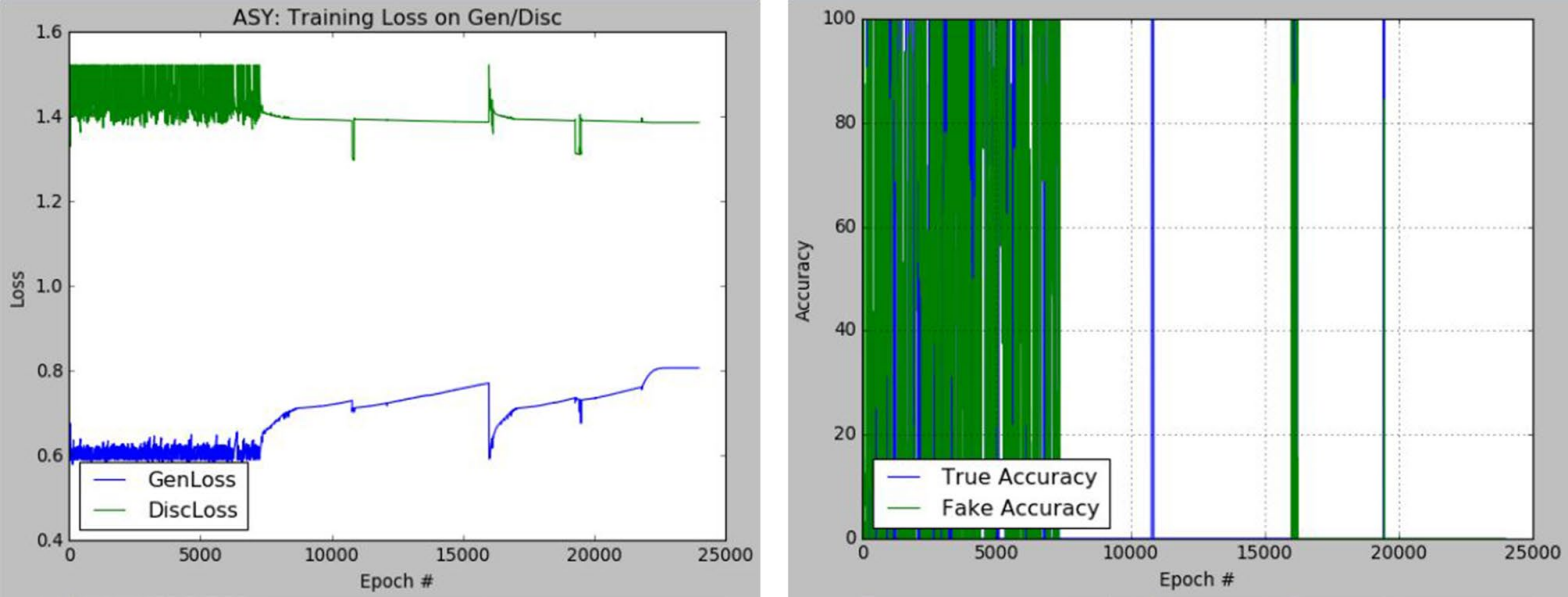
Training output on the proposed GAN model for asymmetry showing how the generator learns in synthesizing images similar to real samples with asymmetry.

**Figure 10:**
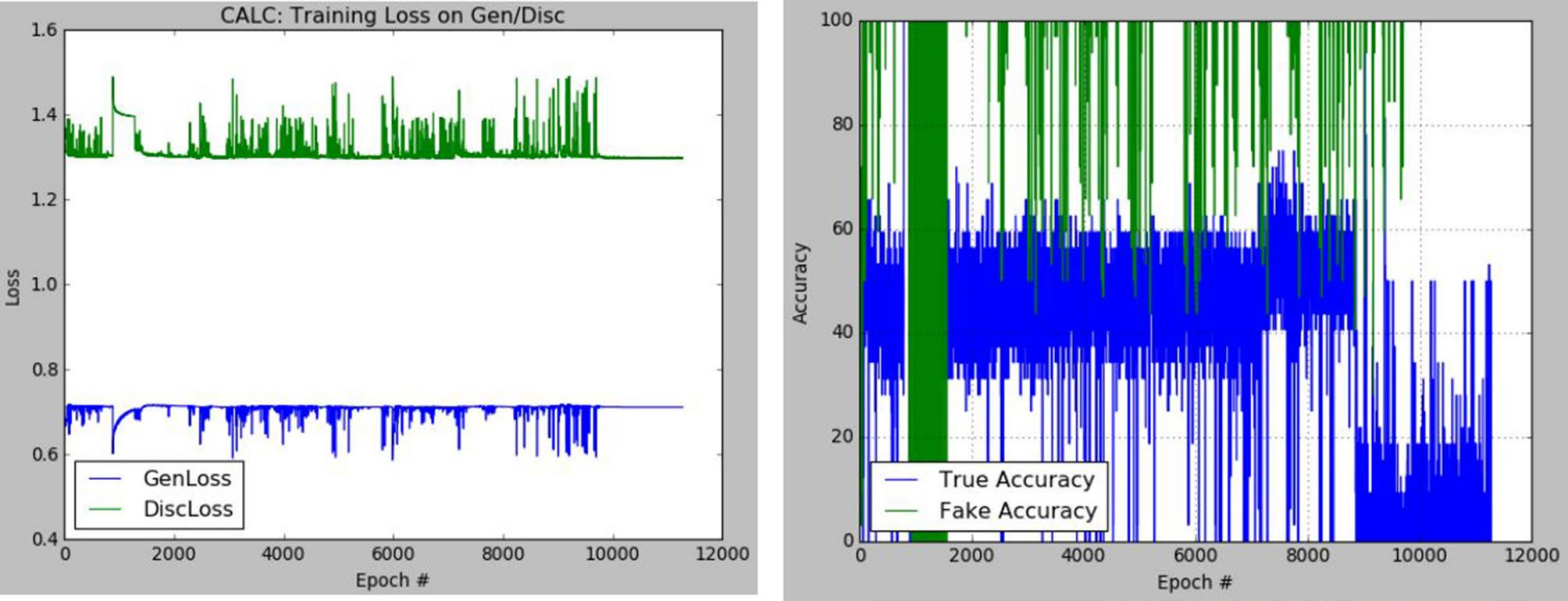
Training output on the proposed GAN model for microclacification showing how the generator learns in synthesizing images similar to real samples with microclacification.

**Figure 11:**
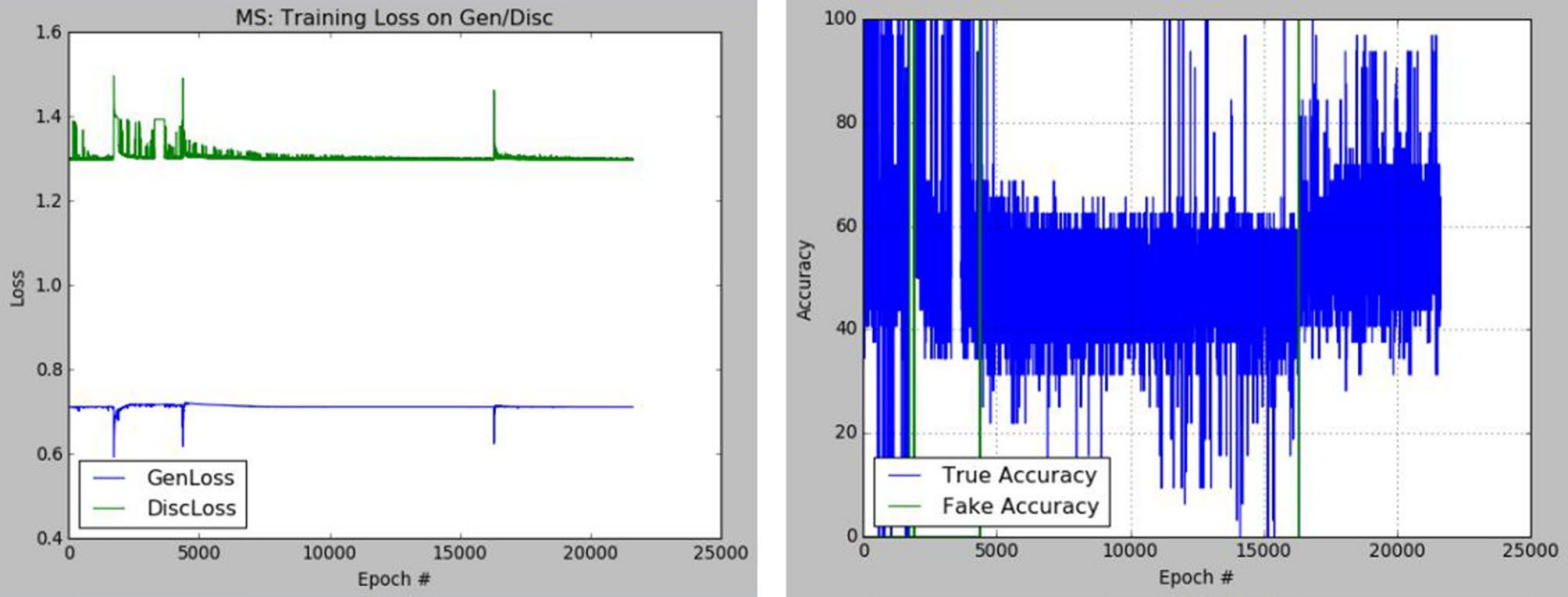
Training output on the proposed GAN model for mass showing how the generator learns in synthesizing images similar to real samples with architectural distortion mass.

The plots of the loss values and accuracies obtained during training for samples with architectural distortion, asymmetry, microclassification, and mass are shown in Figs. 8, 9, 10, and 11, respectively. We found this very interesting to describe how our model learns the problem of generating these images with different abnormalities. The losses for both G and D are collected, and we illustrate the variation in the rise and fall of their losses, respectively.

We observed the training of the GAN model on the samples with architectural distortion and found that the generator is able to generate images with an average accuracy of 85%, as shown in Fig. 8. In the same figure, we observed that the generator's loss values and discriminator rise and fall, respectively. This trend of loss values for both the generator and discriminator also presents similar plots for asymmetry, microcalcification, and mass. The implication of this trend in loss values confirms a progressive and useful learning pattern. Meanwhile, the accuracy of samples generated for the abnormalities architectural distortion, asymmetry, microcalcification, and mass have also been plotted. For instance, we have observed that the model has appeared to be learning quickly to generate samples of asymmetry and microcalcification with significant accuracy, whereas the mass abnormality slowly learns to generate samples with sufficient accuracy. Furthermore, the accuracy of both the true and generated (fake) images was summed, evaluated to 100%, and collected during training. These are computed to determine the significance of the accuracy of both images (real and fake) when combined if they both approach good-quality images.

**Figure 12:**
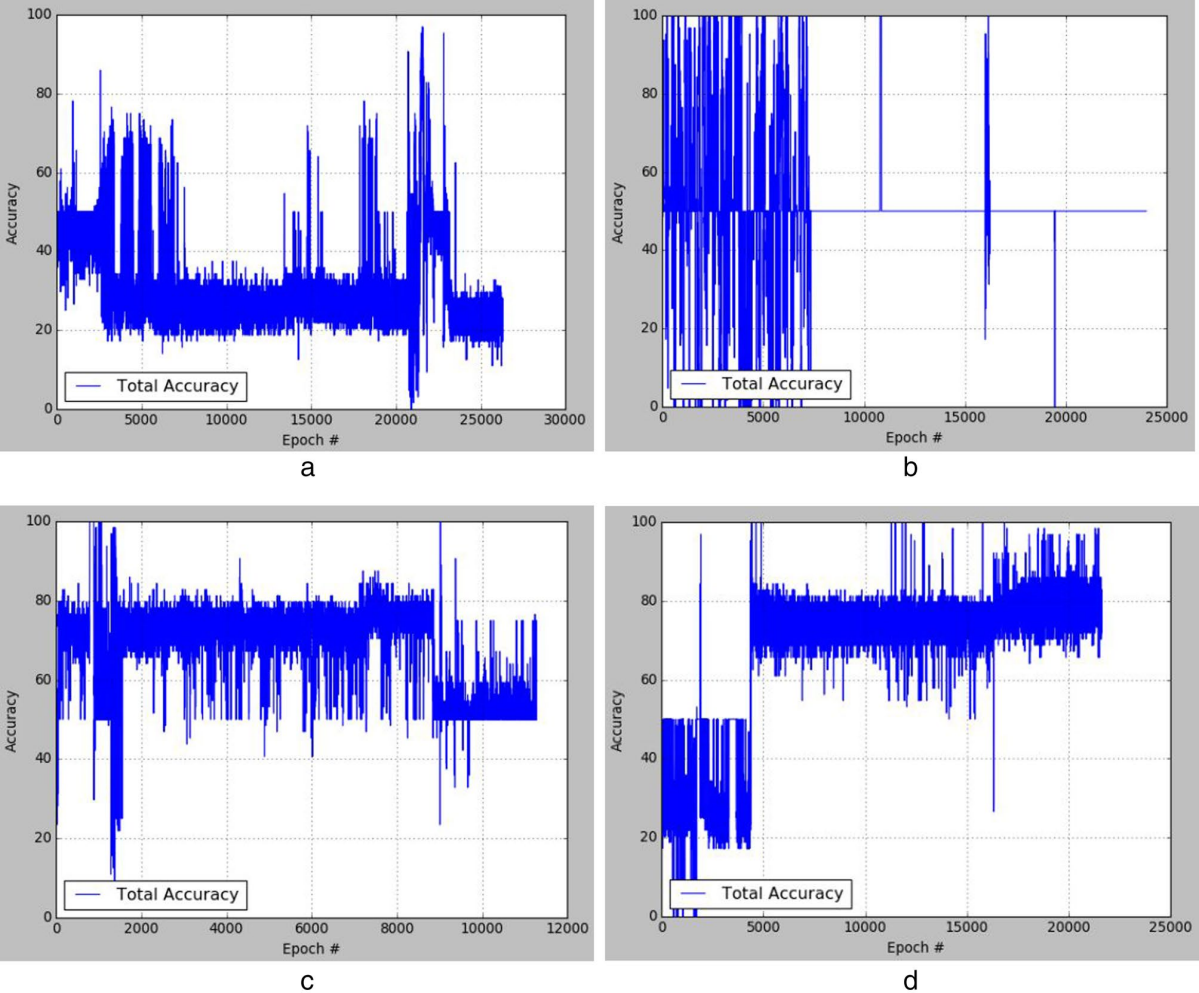
A plot of the combination of the real and generated (fake) accuracies of (**a**) architectural distortion, (**b**) asymmetry, (**c**) microclacification, and (**d**) mass during training as evaluated under 100%.

The outcomes of this analysis are shown in Fig. 12a–c,d for architectural distortion, asymmetry, microclassification, and mass, respectively. This evaluation shows that for samples with architectural distortion, the GAN model improves significantly within the epochs of 20,000–25,000. A similar result has been seen in the cases of microcalcification and mass abnormalities as the accuracy of both fake and real images improved progressively, although the former presents a false performance in the early phase of training. Meanwhile, the discriminator has demonstrated poor performance in learning the samples with asymmetry abnormalities, as the generator is able to fool it in the early phase of training.

**Figure 13:**
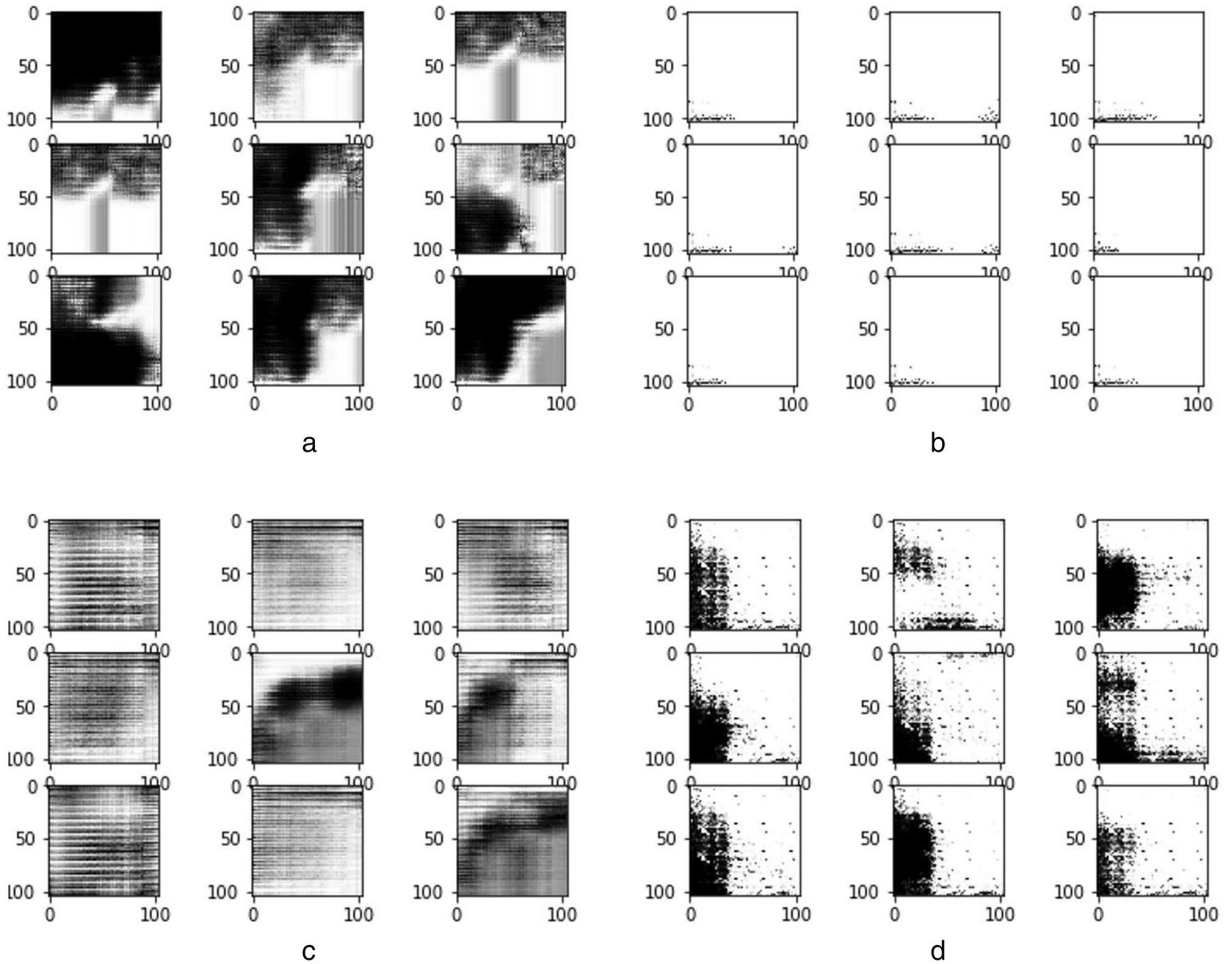
(**a**) Sample images generated during the training of inputs with architectural distortion abnormality. (**b**) Sample images generated during the training of inputs with asymmetrical abnormalities. (**c**) Sample images generated during the training of inputs with microclacification abnormalities. (**d**) Sample images generated during the training of inputs with mass abnormality.

We obtained samples of images generated during the training of the four classes of abnormalities, namely, architectural distortion, asymmetry, microclacification, and mass. Figure 13 shows these samples, demonstrating how the GAN model approaches synthesizing quality ROI-based digital mammograms for those abnormalities. Each case of the abnormalities is captured in Fig. 13a–c,d for architectural distortion, asymmetry, microclassification, and mass, respectively.

**Figure 14:**
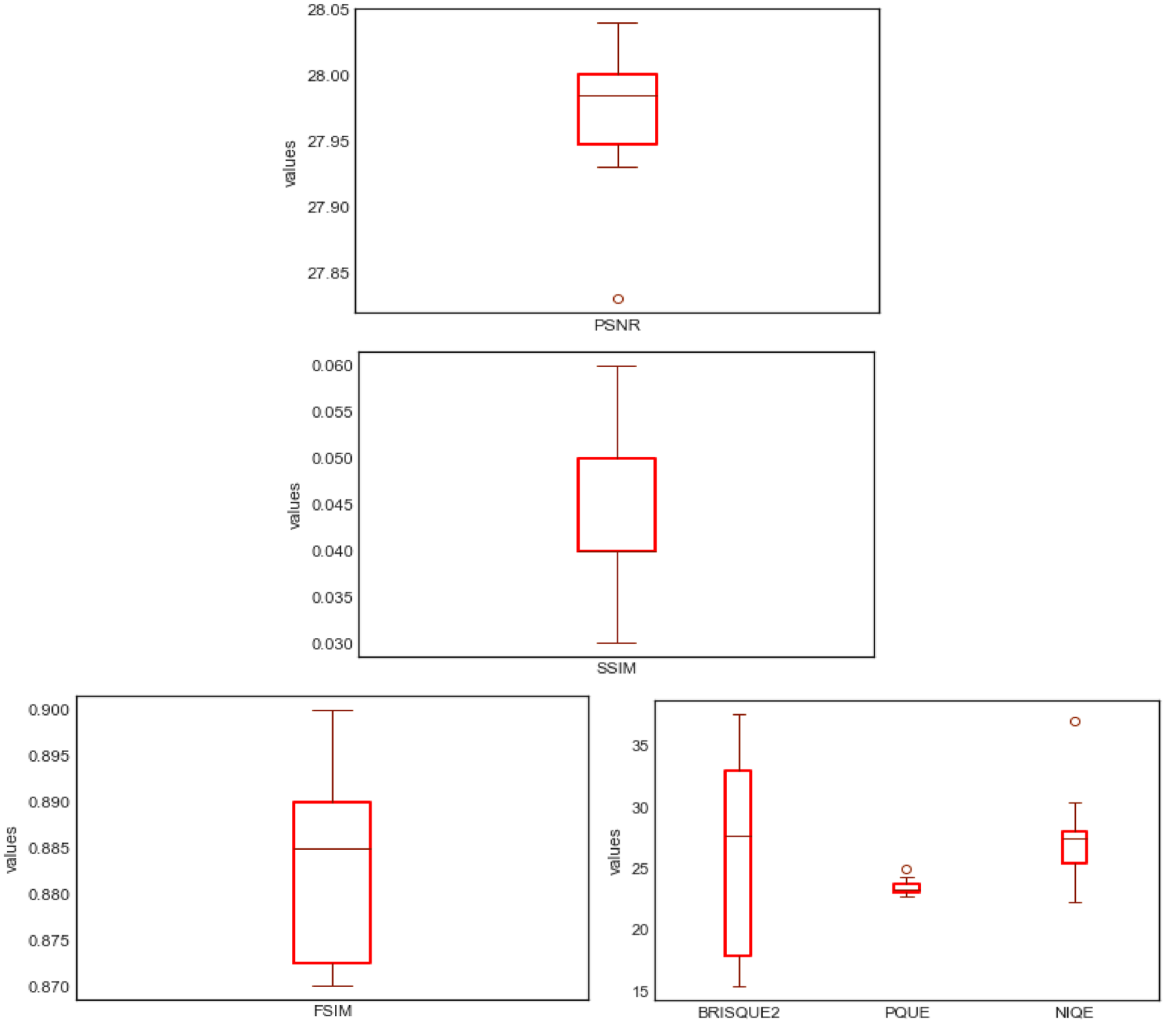
Boxplot showing the distribution of values obtained for ten randomly selected samples of architectural distortion in computational metrics PSNR, SSIM, FSIM, BRISQUE, PQUE, and NIQE.

**Figure 15:**
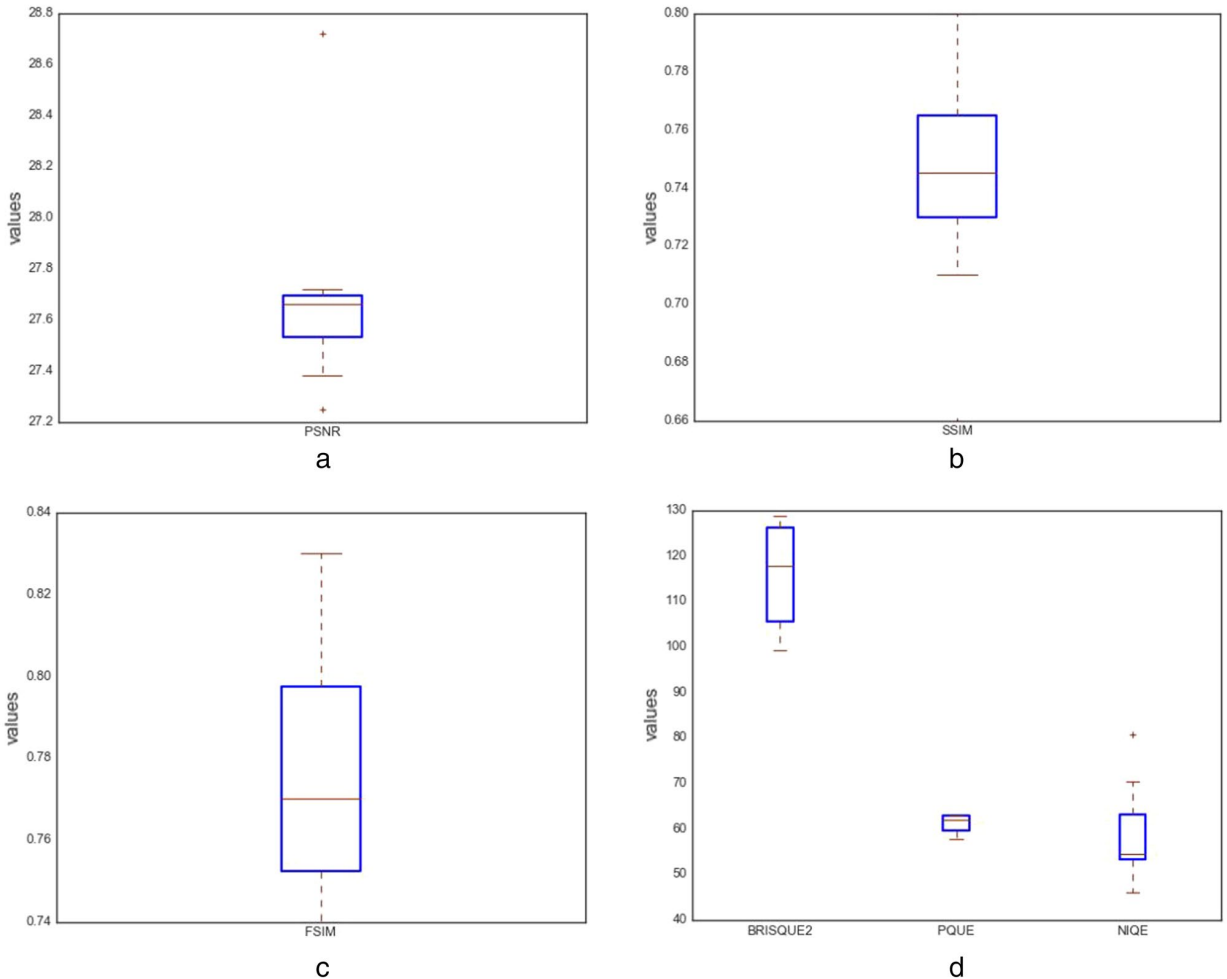
Boxplot showing the distribution of values obtained for ten randomly selected samples of asymmetry in computational metrics PSNR, SSIM, FSIM, BRISQUE, PQUE, and NIQE.

**Figure 16:**
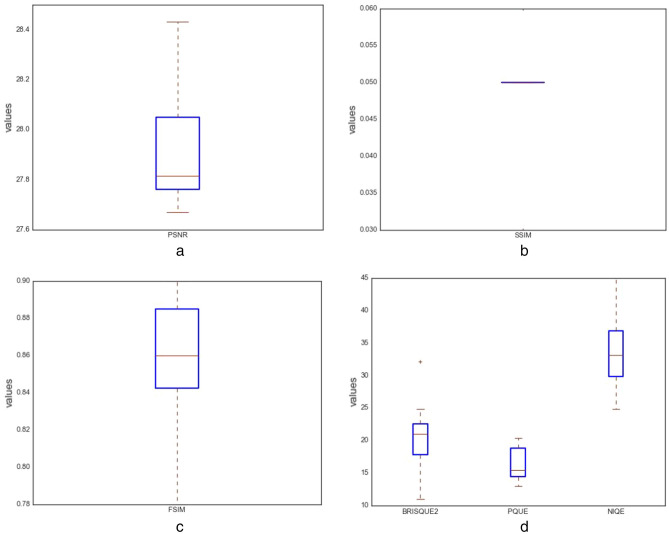
Boxplot showing the distribution of values obtained for ten randomly selected samples of microcalcification in computational metrics PSNR, SSIM, MSE, FSIM, BRISQUE, PQUE, and NIQE.

**Figure 17:**
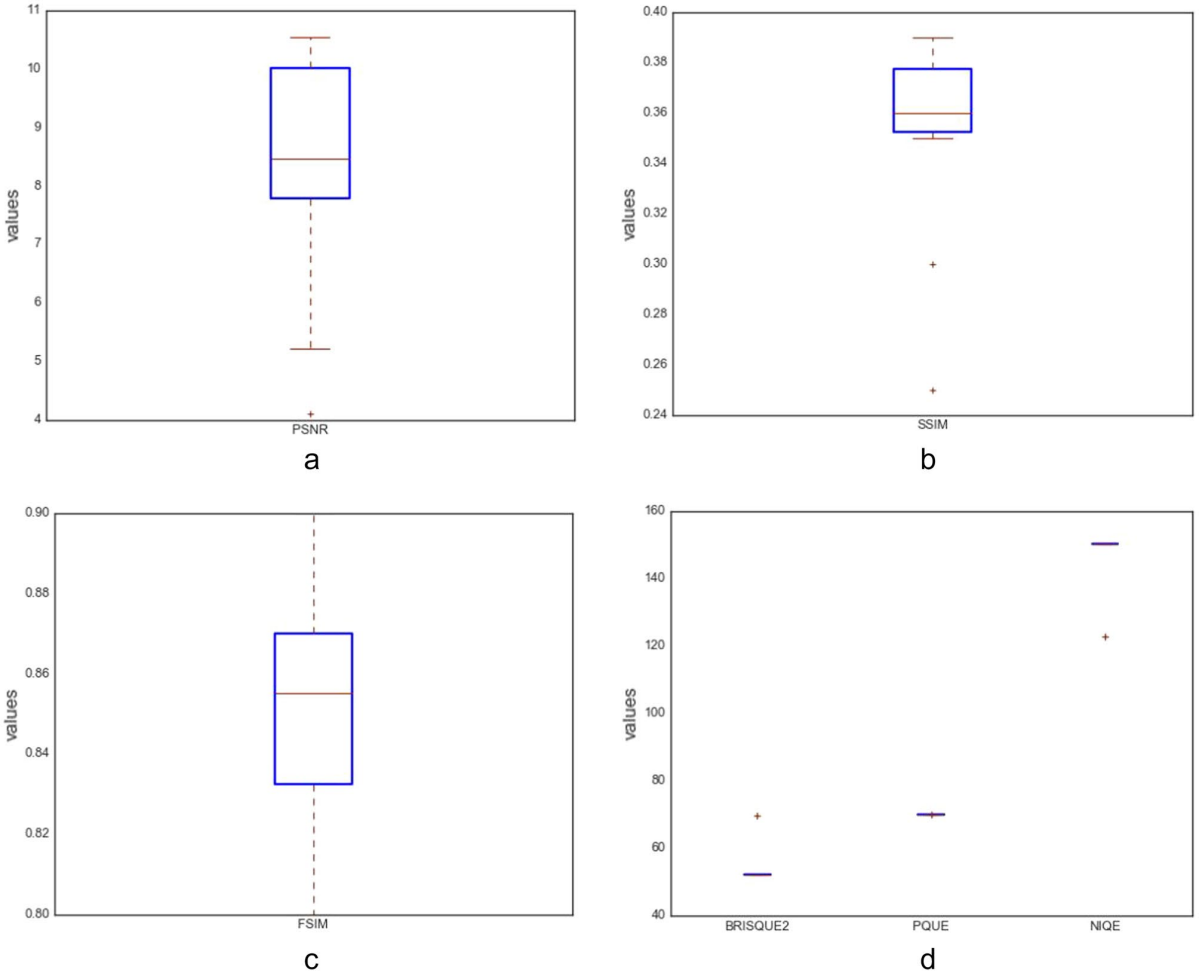
Boxplot showing the distribution of values obtained for ten randomly selected samples of mass in computational metrics PSNR, SSIM, MSE, FSIM, BRISQUE, PQUE, and NIQE.

The state of the models at the epoch, where outputs become qualitative, is saved for synthesizing new samples to support convolutional neural network (CNN) models aimed at classifying abnormalities in digital mammograms. Using these stored models for each abnormality, we generated sample images to apply the computational metrics described in “Performance evaluation metrics” section. In Tables 6, 7, 8, and 9, the values obtained for the PSNR, SSIM, MSE, FSIM, BRISQUE, PQUE, and NIQE metrics are outlined in the cases of architectural distortion, asymmetry, microclassification, and mass, respectively. Ten (10) real samples are drawn from each abnormality and compared with corresponding synthesized samples to confirm and stabilize the analysis. Similarly, to illustrate the distribution of these metrics for each abnormality, we have utilized boxplots in Figs. 14, 15, 16, and 17, which provides good visualization for these distributions.

**Table 6:** Quantitative comparison of the image quality analysis of ten (10) randomly selected synthesized images with architectural distortion (AD) for metrics ranging in the categories of reference-based, nonreference-based, and feature-based.

Images	PSNR	SSIM	DSSIM	MSE	FSIM	BRISQUE	PQUE	NIQE	GS	FID
1	27.97	0.04	0.48	103.73	0.90	27.05	22.65	25.81	0.00	343,130.76
2	28.00	0.04	0.48	103.07	0.88	17.79	23.17	37.07	0.00	321,157.06
3	27.94	0.04	0.48	104.41	0.89	29.11	23.04	30.40	0.00	413,143.07
4	27.83	0.03	0.48	107.18	0.89	15.33	22.70	28.10	0.00	429,359.88
5	27.98	0.04	0.48	103.47	0.89	28.32	23.67	25.31	0.00	330,390.83
6	28.00	0.05	0.48	103.09	0.88	16.62	23.71	27.50	0.00	339,042.88
7	27.93	0.05	0.47	104.61	0.87	34.25	24.23	22.30	0.00	425,196.46
8	27.99	0.04	0.48	103.22	0.87	34.27	23.26	27.56	0.00	308,565.14
9	28.02	0.06	0.47	102.61	0.90	18.04	23.01	27.48	0.00	260,963.64
10	28.04	0.05	0.47	102.22	0.87	37.61	24.91	24.45	0.00	335,339.00
Average	27.97	0.04	0.48	103.76	0.88	25.84	23.44	27.60	0.00	350,628.87

**Table 7:** Quantitative comparison of the image quality analysis of ten (10) randomly selected synthesized images with asymmetry (ASY) for metrics ranging in the categories of reference-based, nonreference-based, and feature-based.

Images	PSNR	SSIM	DSSIM	MSE	FSIM	BRISQUE	PQUE	NIQE	GS	FID
1	27.60	0.74	0.13	112.95	0.77	128.64	59.77	53.52	0.00	261,029.43
2	27.68	0.71	0.15	111.05	0.79	99.24	62.88	53.10	0.00	366,081.45
3	27.25	0.77	0.11	122.58	0.80	106.95	61.86	70.46	0.00	225,235.34
4	27.72	0.80	0.10	109.92	0.76	122.02	62.92	64.81	0.00	206,818.55
5	27.67	0.73	0.14	111.30	0.75	126.44	59.49	54.14	0.00	303,943.84
6	27.38	0.73	0.13	118.84	0.81	126.58	62.47	54.82	0.00	278,837.33
7	28.72	0.66	0.17	87.29	0.83	101.89	57.70	46.08	0.00	380,301.12
8	27.51	0.75	0.13	115.31	0.74	113.56	62.98	50.48	0.00	281,589.40
9	27.65	0.75	0.13	111.68	0.75	125.46	58.68	80.70	0.00	280,155.78
10	27.70	0.79	0.11	110.34	0.77	105.03	61.94	58.10	0.00	221,111.01
Average	27.69	0.74	0.13	111.13	0.78	115.58	61.07	58.62	0.00	280,510.32

**Table 8:** Quantitative comparison of the image quality analysis of ten (10) randomly selected synthesized images with microcalcification (CALC) for metrics ranging in the categories of reference-based, nonreference-based, and feature-based.

Images	PSNR	SSIM	DSSIM	MSE	FSIM	BRISQUE	PQUE	NIQE	GS	FID
1	27.74	0.05	0.48	109.44	0.84	18.63	19.56	34.02	0.00	1,147,952.54
2	28.11	0.05	0.47	100.46	0.86	21.52	16.54	44.94	0.00	1,168,311.50
3	27.75	0.05	0.47	109.27	0.89	22.43	12.99	33.25	0.00	1,608,844.19
4	27.79	0.03	0.48	108.27	0.87	32.13	20.36	38.35	0.00	961,768.18
5	27.67	0.05	0.47	111.16	0.85	17.46	14.22	32.89	0.00	1,186,538.32
6	27.87	0.05	0.48	106.21	0.89	10.95	15.37	24.84	0.00	973,001.32
7	28.36	0.05	0.48	94.89	0.78	13.00	15.14	25.14	0.00	1,368,235.44
8	27.79	0.05	0.47	108.17	0.86	20.40	15.44	31.20	0.00	1,211,028.80
9	28.43	0.05	0.47	93.38	0.79	22.52	13.48	37.85	0.00	1,578,621.76
10	27.84	0.06	0.47	106.80	0.90	24.81	20.36	29.34	0.00	867,607.44
Average	27.93	0.05	0.48	104.81	0.85	20.39	16.35	33.18	0.00	1,207,190.95

**Table 9:** Quantitative comparison of the image quality analysis of ten (10) randomly selected synthesized images with mass (MS) for metrics ranging in the categories of reference-based, nonreference-based, and feature-based.

Images	PSNR	SSIM	DSSIM	MSE	FSIM	BRISQUE	PQUE	NIQE	GS	FID
1	7.61	0.35	0.33	1.13+04	0.84	52.10	69.89	150.22	0.00	446,829.53
2	10.36	0.38	0.31	1.13+04	0.80	52.10	69.89	150.22	0.00	481,668.03
3	10.53	0.39	0.31	1.13+04	0.84	69.52	69.96	122.82	0.00	429,579.98
4	8.97	0.37	0.32	1.13+04	0.83	52.10	69.89	150.22	0.00	462,492.35
5	8.34	0.36	0.32	1.13+04	0.87	52.10	69.89	150.22	0.00	443,899.24
6	8.37	0.36	0.32	1.13+04	0.83	52.10	69.89	150.22	0.00	465,602.81
7	8.54	0.36	0.32	1.13+04	0.87	52.10	69.89	150.22	0.00	497,179.28
8	5.21	0.30	0.35	1.13+04	0.90	52.10	69.89	150.22	0.00	571,124.48
9	4.11	0.25	0.38	1.13+04	0.88	52.10	69.89	150.22	0.00	580,729.34
10	10.54	0.39	0.31	1.13+04	0.87	69.52	69.96	122.82	0.00	448,607.70
Average	8.26	0.35	0.32	1.13+04	0.85	55.59	69.91	144.74	0.00	482,771.27

The results of the reference-based metrics applied to the evaluation of the synthesized images, as listed in Tables 6, 7, and 8, reveal that the MSE, the average squared difference between the real and synthesized images, is minimal compared to what was obtained in Table 9. This indicates that our GAN model can learn the representation of samples from architectural distortion, asymmetry, and microcalcification abnormalities except for some challenges encountered in the case of mass abnormalities. Furthermore, to investigate the images' quality, we compute the PSNR, SSIM, DSSIM, and FSIM metrics. We discovered that for the PSNR metric, average values of 27.97, 27.69, and 27.93 were obtained for architectural distortion, asymmetry, and microcalcifications, respectively, whereas the mass abnormality yielded 8.26 for the same metric. Additionally, for the SSIM and DSSIM metrics, which also evaluate the quality of an image, paired values of 0.04 and 0.48, 0.74 and 0.13, and 0.05 and 0.48 are obtained for architectural distortion, asymmetry, and microcalcifications, respectively, with mass abnormalities of 0.35 and 0.32 for the paired metrics. The performance of the GAN model on the four abnormalities when evaluated with FSIM metrics shows that the average values of 0.88, 0.78, 0.85, and 0.85 were obtained for architectural distortion, asymmetry, microcalcifications, and mass abnormalities, which are very competitive. The outcome of the reference-based metrics implies that the images generated by the proposed GAN model are acceptable. Meanwhile, to demonstrate the distribution of values across the ten (10) samples obtained from the synthesized images for each metric in this category, Figs. 14, 15, 16, and 17 depict their plots for architectural distortion, asymmetry, microcalcifications, and mass abnormalities, respectively. The data in this distribution show that the GAN model's success in generating samples is primarily determined by features learned since just a few outliers in the boxplot are noticed.

Nonreference-based metrics are also evaluated against the proposed GAN model, and the results obtained are listed in Tables 6, 7, 8, and 9 for architectural distortion, asymmetry, microcalcifications, and mass abnormalities, respectively. For instance, the results obtained for the BRISQUE metric reveal that average values of 25.84, 115.58, 20.39, and 55.59 are obtained for architectural distortion, asymmetry, microcalcifications, and mass abnormalities, respectively. The GAN model performed appreciably well in the cases of architectural distortion and microcalcification abnormalities, whereas those of asymmetry and mass abnormalities were trailed behind in performance. Similarly, for the PIQE metrics, average values of 23.44, 61.07, 16.35, and 69.91 are obtained for architectural distortion, asymmetry, microcalcifications, and mass abnormalities, respectively. We see a similar distribution in performance by the proposed GAN model, where both abnormalities of architectural distortion and microcalcifications show better outcomes than those of asymmetry and mass abnormalities. This consistency on the side of the model still confirms that the model is able to maintain the syncretization pattern based on what it learned in the case of each abnormality. Finally, the NIQE metric is also evaluated on the proposed GAN model in all cases of abnormalities, and the results showed that average values of 27.60, 58.62, 33.18, and 144.74 resulted in architectural distortion, asymmetry, microcalcifications, and mass abnormalities, respectively. The distribution of the values for the ten (10) samples of synthesized images in the case of architectural distortion, asymmetry, microcalcifications, and mass abnormalities are plotted in Figs. 14, 15, 16, and 17, respectively, for these three metrics in the category of nonreference based. As reflected in their average values, we see that the distribution of values for the architectural distortion, microcalcifications, and asymmetry for the BRISQUE, PIQE, and NIQE metrics are closely spaced with minimal outliers, while that of the mass abnormalities for the same metrics BRISQUE, PIQE, and NIQE indicated more outliers and wider distribution values.

The feature-based metrics are also evaluated against the GAN model proposed in this study. Particularly, the geometry score (GS) and the FID metrics are evaluated to investigate and compare the feature constitution that exists between the real image and those synthesized by the GAN model. This feature similarity measurement for GS in all cases of architectural distortion, asymmetry, microcalcifications, and mass abnormalities tends toward zero (0), confirming the good visual quality of the images generated by the proposed GAN model and indicating that the images, when compared with those from the real distribution, are almost identical with little diversity in their topology. Similarly, the values obtained for the FID metric, which is a calculation of the distance between the real image and that synthesized, are expected to yield many values. As seen in Tables 6, 7, 8, and 9 for architectural distortion, asymmetry, microcalcifications, and mass abnormalities, respectively, those values obtained are significant for all abnormalities.

**Figure 18:**
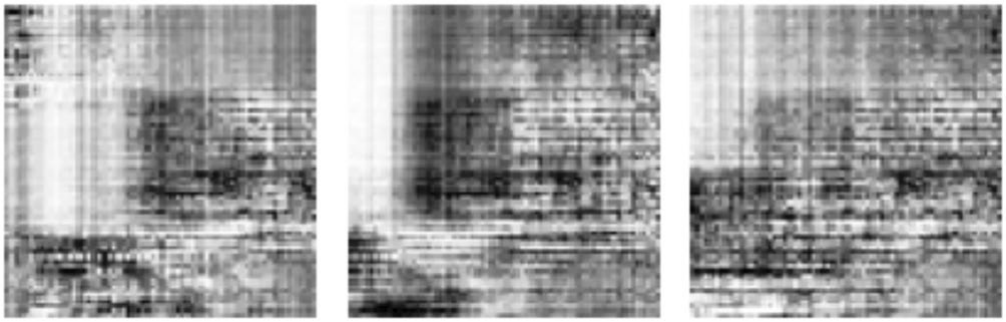
Sample image outputs in 1000 iterations with architectural distortion synthesized using the fully trained generator.

In Fig. 18, sample images synthesized using the fully trained GAN model are presented. These represent regions of interest (ROIs) with different forms of abnormalities.

**Figure 19:**
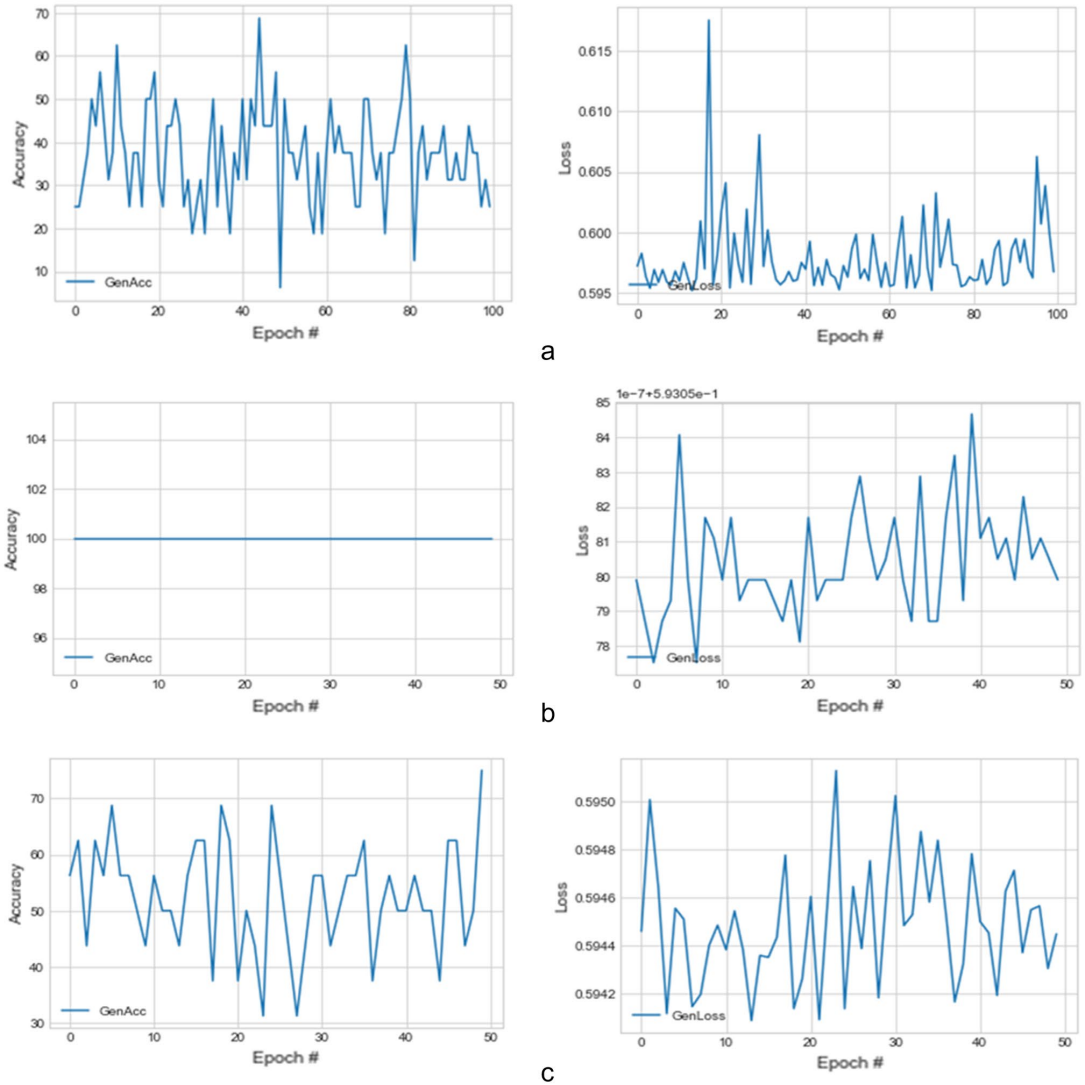
Plot of accuracy and loss values for testing the trained model on samples with (**a**) architectural distortion, (**b**) asymmetry, and (**c**) microcalcification.

**Figure 20:**
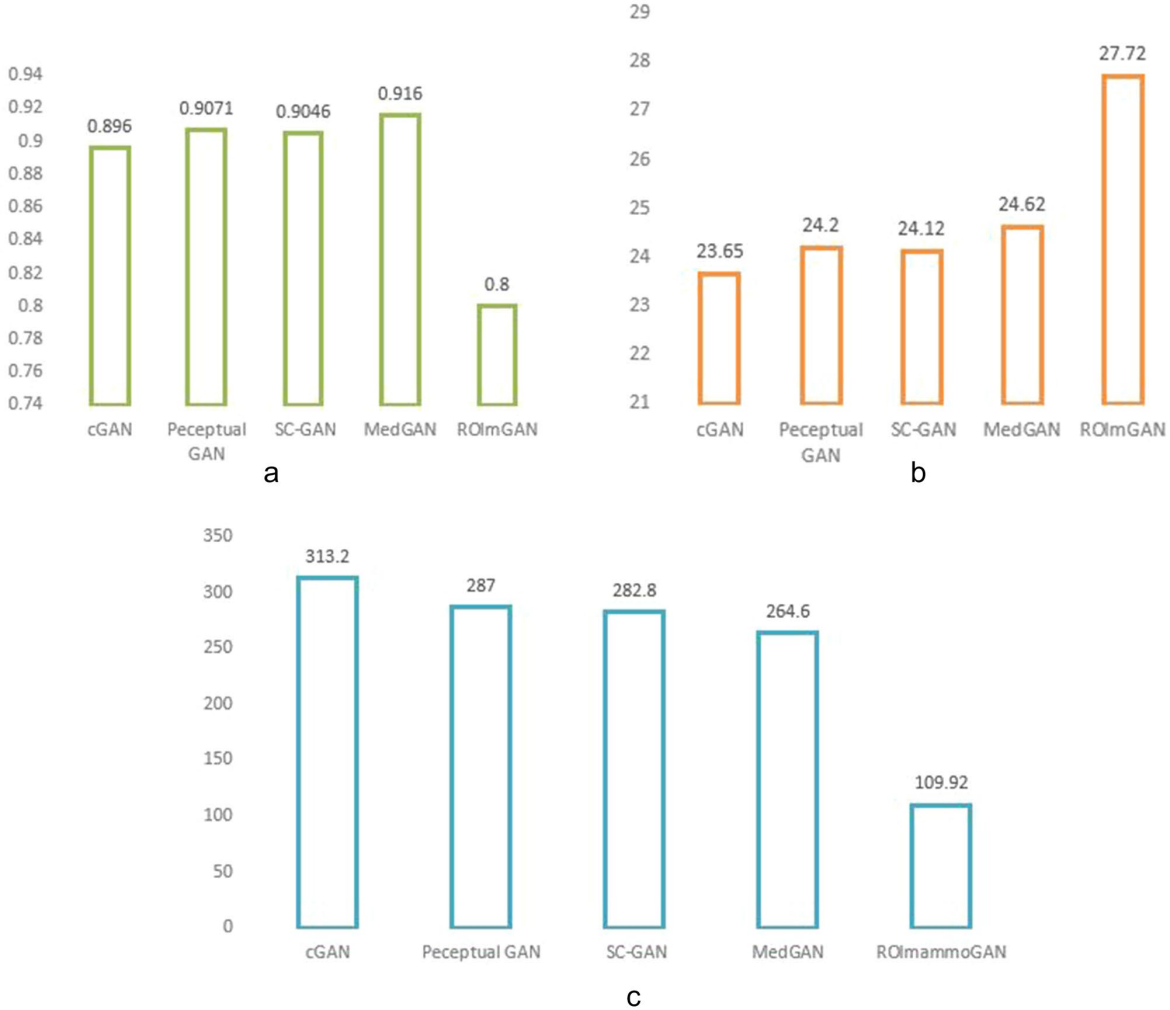
A graphical comparison of the performance of the proposed GAN model in this study compared with similar state-of-the-art medical image GANs: (**a**) SSIM, (**b**) PSNR, and (**c**) MSE.

The results plotted in Fig. 19a–c present the loss values and accuracy obtained for the first fifty (50) samples generated with the trained GAN model in the cases of architectural distortion, asymmetry, and microcalcification abnormalities, respectively. Interestingly, we discovered that the asymmetry accuracy consistently outputs 0.1, while those of architectural distortion and microcalcification peak to approximately 0.7 and 0.78, respectively. To compare the performance of the proposed ROImammoGAN with state-of-the-art image synthesizing models, we carried out a comparative analysis of the work in this study and presented the results in Table 10. A corresponding plot of the results obtained for SSIM, PSNR, and MSE in the table is shown in Fig. 20.

**Table 10:** Comparison of the performance of GAN proposed in this study with state-of-the-art GANs using metrics of reference-based category.

Authors and references	GAN model	SSIM	DSSIM	PSNR	MSE
^69^	Conditional GAN (cGAN)	0.8960	0.05	23.65	313.2
^94^	Peceptual GAN	0.9071	0.05	24.20	287
^56^	Style-content (SC-GAN)	0.9046	0.05	24.12	282.8
^79^	MedGAN	0.9160	0.04	24.62	264.8
This study	ROImammoGAN	0.8000	0.10	27.72	109.92

The comparison of the performance of the proposed ROImammoGAN, as seen in Table 10, reveals that based on the SSIM metric, which evaluates the quality of the real image compared with the synthesized image, our model trails those of cGAN, perceptual GAN, SC-GAN, and MedGAN with values of 0.8960, 0.9071, 0.9046, and 0.9160 compared with our proposed model, which outputs 0.8000. The range of distribution of similar studies and that obtained by our study illustrate that the quality of our generated samples is significant. This comparison is plotted in Fig. 20a. Similarly, we have compared the performance of our model with other state-of-the-art GAN models using the structural dissimilarity SSIM (DSSIM) and demonstrated that the values of 0.05, 0.05, 0.05, and 0.04 obtained by cGAN, perceptual GAN, SC-GAN, and MedGAN compared with 0.10 yielded by our model are good. The PSNR, which also measures the quality of an image with respect to a synthesized image, is also applied to evaluate our GAN model in comparison with similar models. The results show that our GAN model outputs a better performance by attaining a value of 27.72 for PSNR compared with those of 23.65, 24.20, 24.12, and 24.62 cGAN, perceptual G SC-GAN, and MedGAN. A graphical illustration of these values is shown in Fig. 20b. This also confirms that the quality of images synthesized by our proposed GAN model is acceptable and qualitative. Finally, for the MSE metric, we see from Table 10 that our suggested GAN model showed the lowest mean squared error value compared with values obtained from state-of-the-art GAN models used for the comparison task, and the plot in Fig. 20c also confirms this. In summary, the implication of the findings from the results obtained in the experimentation of the proposed ROImammoGAN demonstrates that the model is useful for generating image samples for different abnormalities of digital mammography images. Therefore, the outcome of this study is a GAN model capable of synthesizing ROI-based image samples in the category architectural distortion, asymmetry, microcalcifications, and mass abnormalities. These synthesized images may be used to augment class-imbalanced datasets, which may further be used for classification problems in CNN architectures.

## Conclusion

This study presented a GAN model for generating digital mammograms to augment insufficient publicly and privately available benchmark datasets. The approach adopted in this paper is similar to that of DCGAN, except that training was carried out category-based for abnormalities associated with breast images. First, the discriminator was designed so that it is able to discriminate samples drawn from original data and those synthesized using the generator. The combined G and D models were fine-tuned to learn the characteristics of features in the categories of abnormalities in the databases. Meanwhile, image preprocessing techniques were applied to samples drawn from public datasets to ensure that the proposed GAN model synthesizes good examples. Experimentation was conducted using MIAS benchmark datasets, and the results obtained showed that the proposed GAN model performed well, demonstrating the state-of-the-art GAN models for synthesizing digital mammography images of different abnormalities. Therefore, the resulting GAN model can be adopted for image synthesis and augmentation in studies characterizing abnormalities in breast images. In the future, we aim to investigate the adaptation of the proposed GAN model to produce samples with digital histopathology images and those most interestingly, for 3D tomosynthesis images.

## Data Availability

Freely available MIAS ROI Database that supports the findings of this study was used^88^. Moreover, all methods were carried out in accordance with relevant guidelines and regulations as prescribed by the journal.

## Abbreviations

PSNR: Peak Signal to Noise Ratio
AD: Architectural distortion
AdaGAN: Adaptive GAN
AI: Artificial intelligence
ASY: Asymmetry
BCDR: Breast Cancer Digital Repository
BEGANs: Boundary equilibrium GANs
BRISQUE: Blind/referenceless image spatial quality evaluator
C2ST: Classifier two-sample tests
CALC: Calcification
CBIS-DDSM: Digital Database for Screening Mammography
CC: Craniocaudal
CD: Contrast distortion
ciGAN: Class-conditional GAN
CNN: Convolutional neural network
D: Discriminator
DCGAN: Deep convolutional GAN
DDSM: Digital Database for Screening Mammography
DL: Deep learning
DSSIM: Dissimilarity Structured Similarity Indexing Method
ESRGAN: Enhanced SRGAN
FFDM: Full Field Digital Mammograms
FID: Frechet Inception Distance
FSIM: Feature Similarity Indexing Method
G: Generators
GAM: Generative adversarial metric
GAN: Generative adversarial networks
GD: Gradient descent
GP-GAN: Gaussian-Poisson Generative Adversarial Network
GS: Geometry score
IRMA: Image Retrieval in Medical Applications
IS: Inception Score
LAPGAN: Laplacian GAN
LC: Correlation
LD: Luminance distortion
LLIS: Low-level image statistics
LSGANs: Least Squares Generative Adversarial Networks
MedGAN: Medical GAN
MIAS: Mammographic Image Analysis Society
MLO: Mediolateral-oblique
MMD: Maximum mean discrepancy
MR: Magnetic resonance
MS: Mass
MSE: Mean Square Error
NDB: Number of statistically different bins
NIQUE: Natural Image Quality Evaluator
NNRs: Nonneuro radiologists
NRDS: Normalized relative discriminative score
NRs: Neuro radiologists
PPGNs: Plug and Play Generative Networks
PQUE: Perception based Image Quality Evaluator
ReLU: Rectified linear unit
RGBD: Red, green, blue dimension
RNN: Recurrent neural network
ROIs: Regions of interest
RRDB: Residual-in-Residual Dense Block
S2: Style and Structure
SalGAN: Saliency GAN
SGD: Stochastic gradient descent
SRGAN: Super-Resolution Generative Adversarial Network
SSIM: Structured Similarity Indexing Method
TWRSR: Tournament win rate and skill rating
UIQI: Universal image quality index
UNIT: Unsupervised image-to-image translation model
WGAN: Wasserstein GAN
WGANs: Wasserstein GANs boundary equilibrium GANs
